# Mapping Lipid C=C Isomer Profiles of Human Gut Bacteria through a Novel Structural Lipidomics Workflow Assisted by Chemical Epoxidation

**DOI:** 10.1021/acs.analchem.4c02697

**Published:** 2024-10-22

**Authors:** Kai-Li Chen, Ting-Hao Kuo, Cheng-Chih Hsu

**Affiliations:** † Department of Chemistry, National Taiwan University, Taipei, 10617, Taiwan

## Abstract

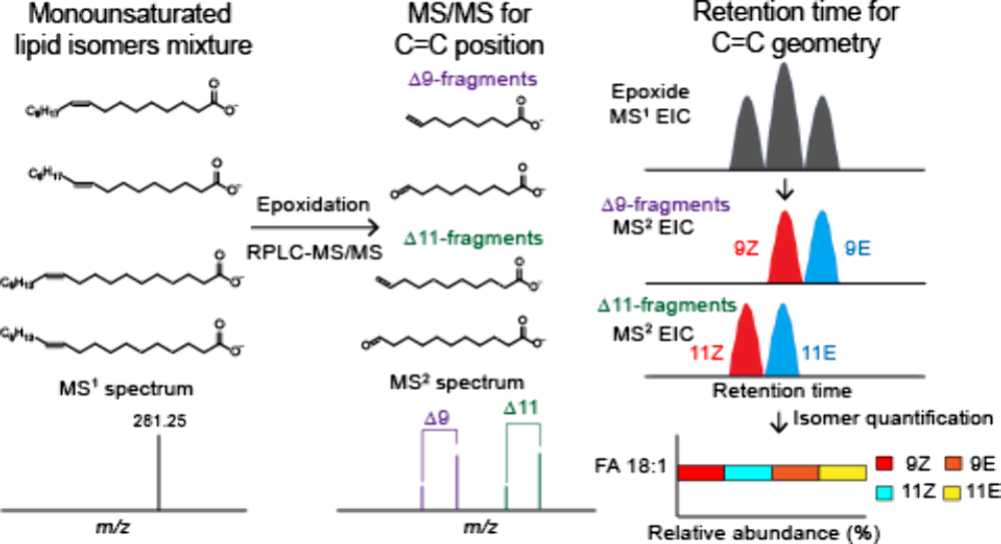

The unsaturated lipids produced by human gut bacteria have an extraordinary range of structural diversity, largely because of the isomerism of the carbon–carbon double bond (C=C) in terms of its position and stereochemistry. Characterizing distinct C=C configurations poses a considerable challenge in research, primarily owing to limitations in current bioanalytical methodologies. This study developed a novel structural lipidomics workflow by combining MELDI (*meta-*chloroperoxybenzoic acid epoxidation for lipid double-bond identification) with liquid chromatography–tandem mass spectrometry for C=C characterization. We utilized this workflow to quantitatively assess more than 50 C=C positional and *cis/trans* isomers of fatty acids and phospholipids from selected human gut bacteria. Strain-specific isomer profiles revealed unexpectedly high productivity of *trans*-10-octadecenoic acid by *Enterococcus faecalis*, *Bifidobacterium longum*, and *Lactobacillus acidophilus* among numerous *trans*-fatty acid isomers produced by gut bacteria. Isotope-tracking experiments suggested that gut bacteria produce *trans*-10-octadecenoic acid through the isomeric biotransformation of oleic acid *in vitro* and that such isomeric biotransformation of dietary oleic acid is dependent on the presence of gut bacteria *in vivo*.

## Introduction

The human gastrointestinal tract harbors a vast microbial community involving trillions of bacterial cells, collectively known as the gut microbiota.^1^ These bacteria actively engage in metabolic activities, producing a wide array of small-molecule chemical products, called metabolites, that profoundly influence various aspects of human physiology.^2−6^ The complex metabolic processes that occur within the gut bacterial community involve biochemical interactions with both ingested compounds^7^ and the human host^8^ and bacterial cellular *de novo* synthesis.^9^ Over the preceding decade, extensive characterization of gut bacterial metabolites has revealed a diverse range of functional roles, including immune responses,^10^ disease biomarkers,^11^ and neurological signaling.^12^ Moreover, recent research on biotransformation, one of the mechanisms through which these metabolites are produced, has elucidated the overarching effect of gut bacterial metabolism on drug availability and efficacy.^13^ The diversity of gut bacterial metabolites and the significance of gut biotransformation have led to a thriving area of research that has elucidated the intricate connection between gut bacterial metabolism and human health.

Gut bacterial lipids are crucial bacterial metabolites in the gut. These lipids play various roles in the interaction between gut bacteria and the human host,^14^ including energy homeostasis^15^ and the regulation of inflammation.^16^ Among these lipid metabolites, unsaturated lipids (i.e., lipid metabolites containing carbon–carbon double bonds [C=C] on their fatty acyl chains) are a primary class of lipid metabolites with a diverse structure and diverse biological functions.^17^ Unsaturated lipids are produced from bacteria through *de novo* synthesis and biotransformation.^18,19^ Unlike mammalian unsaturated lipids, little is known about the nature of the chemical structure and biological function of gut bacterial unsaturated lipids, possibly because of the diversity of their structure and the lack of clarity regarding their biotransformation pathway. Accordingly, characterizing gut bacterial unsaturated lipid structures, including the C=C position and geometry, could be key to investigating the function and activities of these metabolites.

In recent years, mass spectrometry (MS) has become a powerful tool in lipidomics because of its ability to provide high-throughput, highly sensitive, and rapid analysis of lipid molecules in biological samples.^20,21^ However, structural information related to unsaturated lipids—especially C=C position and geometry, which are key factors determining the structure, chemical properties, and bioactivity of such lipids^7,22−26^—cannot be obtained through conventional tandem mass spectrometry (MS/MS).^20,27^ To address this challenge, several MS-based methods have been developed. For example, electron transfer dissociation,^28^ electron impact excitation of ions from organics,^29^ ultraviolet photodissociation,^30^ hydrogen abstraction dissociation,^31^ and radical-directed dissociation^32^ all incorporate special fragmentation methods to break down C=C bonds in order to identify C=C position on the basis of the MS^*n*^ spectrum. Other methods of coupling the derivatization of C=C double bonds with MS—including covalent adduct chemical ionization,^33^*N*-(4-aminomethylphenyl) pyridinium derivatization,^34^ Paternò–Büchi reaction,^35^ epoxidation,^36,37^ ozonolysis,^38,39^ aziridination,^40^ and thiol–ene reaction^41^—transform the C=C structure into other, less stable structures so that the C=C position can be determined on the basis of MS^*n*^ spectra of the products. Nonetheless, in these methods, the C=C geometry is still not resolved effectively, because of the similar MS^*n*^ spectra of geometric isomers. To identify geometric isomers, coupling MS with separation methods such as chromatography^42^ and ion mobility^43,44^ is one potential solution. For example, gas chromatography–MS can separate C=C isomers of fatty acid (FA) methyl esters and identify them by retention time.^45^ In addition, Xie and Xia identified C=C geometric isomers in conjugated FAs through trapped ion mobility MS, determining the C=C geometry on the basis of the drift time.^46^ Recently, Feng et al. established a method that combines a photocycloaddition–photoisomerization reaction with liquid chromatography (LC)-MS/MS; this method enables the characterization of either C=C position or C=C geometry without lipid standards.^47^ However, these methods may face considerable challenges in defining both the C=C position and the C=C geometry for multiple unsaturated lipid species in a routine lipidomics experiment.

Our previous study presented *meta-*chloroperoxybenzoic acid (*m*CPBA) epoxidation for lipid double-bond identification (MELDI)^36^ as a method of C=C position identification. In the present study, to utilize the ability of this method to characterize the C=C position while obtaining information regarding C=C geometry, we combined MELDI with reversed-phase LC (RPLC)-MS/MS (RPLC-MS/MS) to yield MELDI-RPLC-MS/MS. Technically, *m*CPBA epoxidation facilitates the characterization of the C=C position, and C=C geometry is determined on the basis of the retention time in a routine lipidomics experiment. The quantitative and qualitative capabilities of MELDI-RPLC-MS/MS were validated on the basis of authentic chemical standards for lipid isomers. To demonstrate its utility, we applied the MELDI-RPLC-MS/MS method to investigate lipid extracts from both human gut bacteria and mice feces. Notably, we observed gut bacteria-related *cis*–*trans* isomerization and demonstrated the effect of gut bacteria on C=C isomerism.

## Experimental Section

### Chemicals and Reagents

FA standards were purchased from Cayman Chemical (Michigan, USA), except FA18:1 (10E) was purchased from Life Lipid (California, USA). Glycerophospholipid (GPL) standards were purchased from Avanti Polar Lipids (Alabaster, AL, USA). Methanol was purchased from Duksan Pure Chemical (Seonggok-dong, Korea), and methyl *tert*-butyl ether (MTBE) was purchased from Alfa Aesar (Massachusetts, USA). *m*CPBA and ammonium acetate were purchased from Sigma-Aldrich (St. Louis, MO, USA). 2-Propanol (IPA) was purchased from Honeywell (Michigan, USA). Acetonitrile (ACN) was purchased from J.T. Baker (New Jersey, USA). Ultrapure water (18.2 M Ω) was prepared by a PureLab Classic system (ELGA, Buckinghamshire, UK). Tryptic soy agar (TSA) with 5% sheep blood agar plate was purchased from Dr. Plate (Taipei, Taiwan). MRS medium was purchased from Neogen (Michigan, USA).

### Lipid Nomenclature

The lipid nomenclature reported by Liebisch et al. was adopted in the study.^48^ FA 18:1 (9Z) represents a fatty acid with 18 carbon atoms and one double bond on the ninth carbon in the *Z*-configuration. The double-bond position was annotated according to the Δ-nomenclature if the configuration was yet to be characterized (e.g., FA18:1 (Δ9)). For the phospholipid, a slash was used when the sn-position of acyl chains was identified (e.g., PG 18:0/16:0), whereas an underscore was used when the sn-position of acyl chains was unknown (e.g., PG 18:0_16:0).

### Protocol of Lipid C=C Derivatization by *m*CPBA

The lipid derivatization reagent was prepared by dissolving 44.8 mg of *m*CPBA powder in 1 mL of methanol to reach a final *m*CPBA concentration of 200 mM. A lipid sample (lipid standard or biological extract) was added with an equal volume of the derivatization reagent, incubated for 1 h at 50 °C, and analyzed by mass spectrometry. The epoxidation protocol was modified from our previous study.^36^

### Instrumental Parameters

All LC-MS experiments were performed using an Orbitrap Elite mass spectrometer (Thermo Scientific, Massachusetts, USA) coupled with a Thermo Vanquish UHPLC (Thermo Scientific). A heated electrospray ionization (HESI) probe was equipped as the ionization source with the following parameters: spray voltage at 3.5 kV in negative ionization mode; capillary temperature at 320 °C; HESI heater temperature at 250 °C; sheath gas flow at 25 (a.u.) and auxiliary gas at 10 (a.u.); the ion optics were tuned at *m*/*z* 283.26 ([M – H]^−^ ion of FA 18:0). The liquid chromatography was performed using a 1.7 μm Waters Acquity CSH C18 column (100 × 2.1 mm, Waters, Massachusetts, USA). A binary gradient was performed with mobile phase A of ACN/water (40/60, v/v) and mobile phase B of IPA/ACN (90/10, v/v). Both A and B solvents contained 10.0 mM ammonium acetate. The optimized 30 min gradient was established as 0–3 min, 20% B; 3–10 min, 20–30% B; 10–15 min, 30–50 % B; 15–21 min, 50–99% B; 21–24 min 99% B; 24–26 min, 99–20% B, 26–30 min, 20% B.

### Targeted Lipid Analysis of the Lipid Standards

A lipid standard mixture containing 14 monounsaturated fatty acid C=C isomers was prepared. The standards used included FA 16:1 (7Z), FA 16:1 (9Z), FA 16:1 (9E), FA 16:1 (11Z), FA 16:1, (11E), FA 18:1 (6Z), FA 18:1 (6Z), FA 18:1 (8Z), FA 18:1 (9Z), FA 18:1 (9E), FA 18:1 (11Z), FA 18:1 (11E), FA 20:1 (11Z), and FA 20:1 (11E). The concentration of each isomer ranged from 0.1 μM to 10 μM. Then the mixture was epoxidated by the addition of an equal volume of 200 mM *m*CPBA. For targeted unsaturated fatty acid C=C isomer analysis, a targeted MS/MS method was established. The scanning cycle consists of one full FT-MS scanning with a mass range of 200–2000 and spectral resolution of 60 000, followed by 5 targeted IT-MS/MS scanning events. The MS/MS spectra of 5 targeted epoxy-precursor ions, FA 16:1 (*m*/*z* = 269.20), FA17:1 (*m*/*z* = 283.30), FA 18:1 (*m*/*z* = 297.24), FA 19:1 (*m*/*z* = 311.26), and FA 20:1 (*m*/*z* = 325.28), were acquired via an ion activation type of CID, isolation width of *m*/*z* 2.0, normalized collision energy (NCE) of 40.0, activation Q of 0.250, and activation time of 10.00 ms. The maximum injection time for both full FT-MS and IT MS^*n*^ was set at 500 ms with automatic gain control (AGC) of 1.00e+6 for full FT-MS scans and 3.00e+4 for IT MS/MS. The data were collected with Xcalibur 3.0 (Thermo Scientific).

### Targeted Unsaturated Fatty Acid C=C Isomer Analysis of the Gut Bacterial Lipid Extracts

For targeted unsaturated fatty acid C=C isomer analysis, a targeted MS/MS method was established. The scanning cycle consists of one full FT-MS scanning with a mass range of 100–1000 and spectral resolution of 60 000, followed by 5 targeted IT-MS/MS scanning events. The MS/MS spectra of 5 targeted epoxy-precursor ions, FA 16:1 (*m*/*z* = 269.21), FA17:1 (*m*/*z* = 283.22), FA18:1 (*m*/*z* = 297.24), FA19:1 (*m*/*z* = 311.25), and FA20:1 (*m*/*z* = 325.27), were acquired via an ion activation type of CID, isolation width of *m*/*z* 1.0, NCE of 40.0, activation Q of 0.250, and activation time of 10.00 ms. The maximum injection time for both full FT-MS and IT MS^*n*^ was set at 500 ms with an AGC of 1.00e+6 for full FT-MS scans and 3.00e+4 for IT MS/MS. The data were collected with Xcalibur 3.0 (Thermo Scientific).

### Gut Bacterial Isotope Tracking Analysis

A targeted MS/MS method was established for gut bacterial isotope tracking analysis. The scanning cycle consists of one full FT-MS scanning with a mass range of 100–1000 and spectral resolution of 60 000, followed by 6 IT-MS/MS scanning events. The MS/MS spectra of 6 targeted epoxy-precursor ions, FA 16:1 (*m*/*z* = 269.25), FA 17:1 (*m*/*z* = 283.25), FA 18:1 (*m*/*z* = 297.80), FA 19:1 (*m*/*z* = 311.30), FA 20:1 (*m*/*z* = 325.30), and D_17_-FA 18:1 (*m*/*z* = 314.30), were acquired via an ion activation type of CID, isolation width of *m*/*z* 2.0, NCE of 40, activation Q of 0.250, and activation time of 10.00 ms. The maximum injection time for both full FT-MS and IT MS^*n*^ was set at 500 ms with an AGC of 1.00e+6 for full FT-MS scans and 3.00e+4 for IT MS/MS. The data were collected with Xcalibur 3.0 (Thermo Scientific).

### *In Vivo* Isotope Tracking Analysis

A targeted MS/MS method was established for gut bacterial isotope tracking analysis. The scanning cycle consists of one full FT-MS scanning with a mass range of 100–1000 and spectral resolution of 60 000, followed by 6 IT-MS/MS scanning events. The MS/MS spectra of 6 targeted epoxy-precursor ions, FA 16:1 (*m*/*z* = 269.25), FA 17:1 (*m*/*z* = 283.25), FA 18:1 (*m*/*z* = 297.24), ^13^C_5_-FA 18:1 (*m*/*z* = 302.25), FA 19:1 (*m*/*z* = 311.30), FA 20:1 (*m*/*z* = 325.30), and D_17_-FA 18:1 (*m*/*z* = 314.30), were acquired via an ion activation type of CID, isolation width of *m*/*z* 2.0, NCE of 40, activation Q of 0.250, and activation time of 10.00 ms. The maximum injection time for both full FT-MS and IT MS^*n*^ was set at 500 ms with an AGC of 1.00e+6 for full FT-MS scans and 3.00e+4 for IT-MS/MS. The data were collected with Xcalibur 3.0 (Thermo Scientific).

### C=C Isomer Quantification

For the quantification of lipid C=C isomers, the fraction of a C=C isomer was calculated by dividing the EIC peak area of the diagnostic ions from a specific C=C isomer by the sum of the EIC peak area of the diagnostic ions from each C=C isomer.

The fraction .

### Calibration Curve of FA 18:1 9Z and 9E Isomers

The total concentration of FA18:1 was kept at 1 μM with the molar ratio varied ([9E]/[9Z] = 99/1, 90/10, 50/50, 10/90, and 1/99). Each mixture was derivatized with excess *m*CPBA (20 mM) at 50 °C for 1 h and was then analyzed by LC-MS. The calibration curve was constructed by plotting the molar fraction of 9E isomers ([9E]/([9E] + [9Z])%) against the fractions of the summed extracted ion chromatogram (EIC) area of diagnostic ions , where , , both obtained from the MS/MS channel at *m*/*z* 297.24. Each point represents a technical triplicate.

### Bacteria Culture

The bacteria used in this study were purchased from the Bioresource Collection and Research Center (BCRC) in Taiwan. The medium for each gut bacteria is listed in Supplementary Table 1. For broth culture, 0.5 mL of stock solution was diluted by 10 mL of medium and grew to a stationary phase before lipid extraction. For agar plate culture, the 0.1 mL of stock solution was transferred to the agar plate and then grew for 3 days before lipid extraction. The whole process was conducted in an anaerobic workstation (N_2_:H_2_:CO_2_ = 8:1:1) (Whitley DG250, Don Whitley Scientific Limited, England).

### *In Vitro* Isotope Tracking

When the bacteria entered the stationary phase, the bacteria solution was transferred to a 15 mL centrifuge tube and was diluted to O.D. ≈ 0.1–0.2 at 600 nm wavelength by addition of MRS broth/MRS broth with 0.5% EtOH/MRS broth with 0.18 mM ^13^C_1_-oleic acid and 0.5% EtOH. ^13^C_1_-Oleic acid was filtered with a 0.22 μm filter (Millex GS filter unit, Merck Millipore, Massachusetts, USA) before spiking into MRS. Each condition was tested in quadruplicate, the 0.500 mL bacteria solution was taken out at different growth times (0, 5, 10, and 24 h), and 0.1 mL of D_17_-FA18:1 (9Z) (10 μM in ethanol) was added as the internal standard. Then 0.600 mL of MTBE and 0.150 mL of MeOH were added to the samples and sonicated for 30 min. The organic layer was transferred to another centrifuge tube, and then 0.3 mL of MTBE and 0.1 mL of MeOH were added for the second extraction. After being sonicated for 10 min, the organic layer was combined and dried in a vacuum concentrator. The 0.1 mL reconstituted solution (ACN/IPA/H_2_O = 65/30/5) was added to dissolve the extracts. Then the samples were epoxidated and analyzed by RPLC-MS/MS.

### Extraction of Gut Bacteria Lipids

The lipid extraction protocol was adapted from the method presented by Vitali et al.^49^ For bacteria growing on an agar plate, the bacterial cells were first scratched into a 1.5 mL microcentrifuge tube by a cell scraper, and then 750 μL of MTBE/MeOH (v/v = 4/1) was added and vortexed for 30 min. Then 200 μL of ddH_2_O was added for phase separation. The sample was then centrifuged for 3 min at 12 000 rpm, and then the upper organic layer was transferred into another 1.5 mL microcentrifuge tube. The second extraction was conducted by adding 100 μL of ddH_2_O, 100 μL of MeOH, and 300 μL of MTBE and vortexed for 10 min. Then the sample was centrifuged at 12 000 rpm for 1 min. After centrifuging, the entire extraction was collected together. The solution was dried in a vacuum concentrator, and then, 0.1 mL of reconstituted solution (ACN/IPA/H_2_O = 65/30/5) was added to dissolve the extracts. For bacteria growing in broth, 0.5 mL of bacteria solution was taken out and 0.1 mL of D_17_-FA 18:1 (9Z) (33 μM in MeOH) was added into the solution. Then 400 μL of MTBE was added, and the solution was sonicated for 30 min. Then the organic layer was transferred into another 1.5 mL microcentrifuge tube. The second extraction was conducted by adding 100 μL of MeOH and 300 μL of MTBE and then sonicated for 10 min. Then the organic layers were combined, dried, and reconstituted as described above. The reconstituted samples were stored at −80 °C before further analysis.

### MUFA Profiling of Gut Bacteria Lipid Extracts

The *m*CPBA reagent (200 mM) was prepared beforehand by dissolving the *m*CPBA powders with MeOH. The lipid extracts were mixed with *m*CPBA at equal volume, and then the mixture was heated at 50 °C for 1 h. The mixture was then analyzed by RPLC-MS/MS, and the fraction of each isomer was calculated by analyzing the corresponding diagnostic ions.

### *In Vivo* Isotope Tracking

To assess the effect of gut bacteria on fatty acid isomers *in vivo*, five 10-week-old female C57BL/6JNarl specific-pathogen-free (SPF) mice and five 12-week-old female C57BL/6JNarl germ-free (GF) mice were orally administrated with the isotope tracer, ^13^C_5_-FA 18:1 9Z (2.7 mg/mL grapeseed oil) at a dose of 20 mg per kilogram of body weight. Mouse fecal pellets were collected before and 6 h after the intake of the isotope tracer respectively for lipid extraction (IACUC number: NLAC-107-O-006-R7). The mouse experiment was done by Leeuwenhoek Laboratories Co. Ltd., Taipei, Taiwan (IACUC number: 00169). The feces were lyophilized, ground into powder, and weighed before lipid extraction. Then the internal standard D_17_-FA 18:1 9Z (33 μM in ethanol) was added at 4.95 μL per milligram of feces. The first extraction was conducted by the addition of MTBE/MeOH (v/v = 4/1) at 37.1 μL per milligram of feces and sonicated for 1 h. Then ddH_2_O was added at 9.9 μL per milligram of feces for phase separation, and the samples were centrifuged at 12 000 rpm for 5 min. The upper organic layer was collected in a centrifuged tube, and ddH_2_O/MeOH/MTBE (v/v/v = 1/1/3) was added into the water layer at 24.8 μL per milligram of feces for the second extraction. The samples were sonicated for 30 min and then centrifuged at 12 000 rpm for 5 min. The organic layers were combined and dried in the vacuum concentrator. Then the reconstituted solution (ACN/IPA/H_2_O = 65/30/5, v/v/v) was added at 4.95 μL per milligram of feces. The reconstituted samples were stored at −80 °C before further analysis.

## Results and Discussion

### Working Principle of MELDI-RPLC-MS/MS for Structural Lipidomics in the Characterization of Lipid C=C Position and Geometry

**Figure 1:**
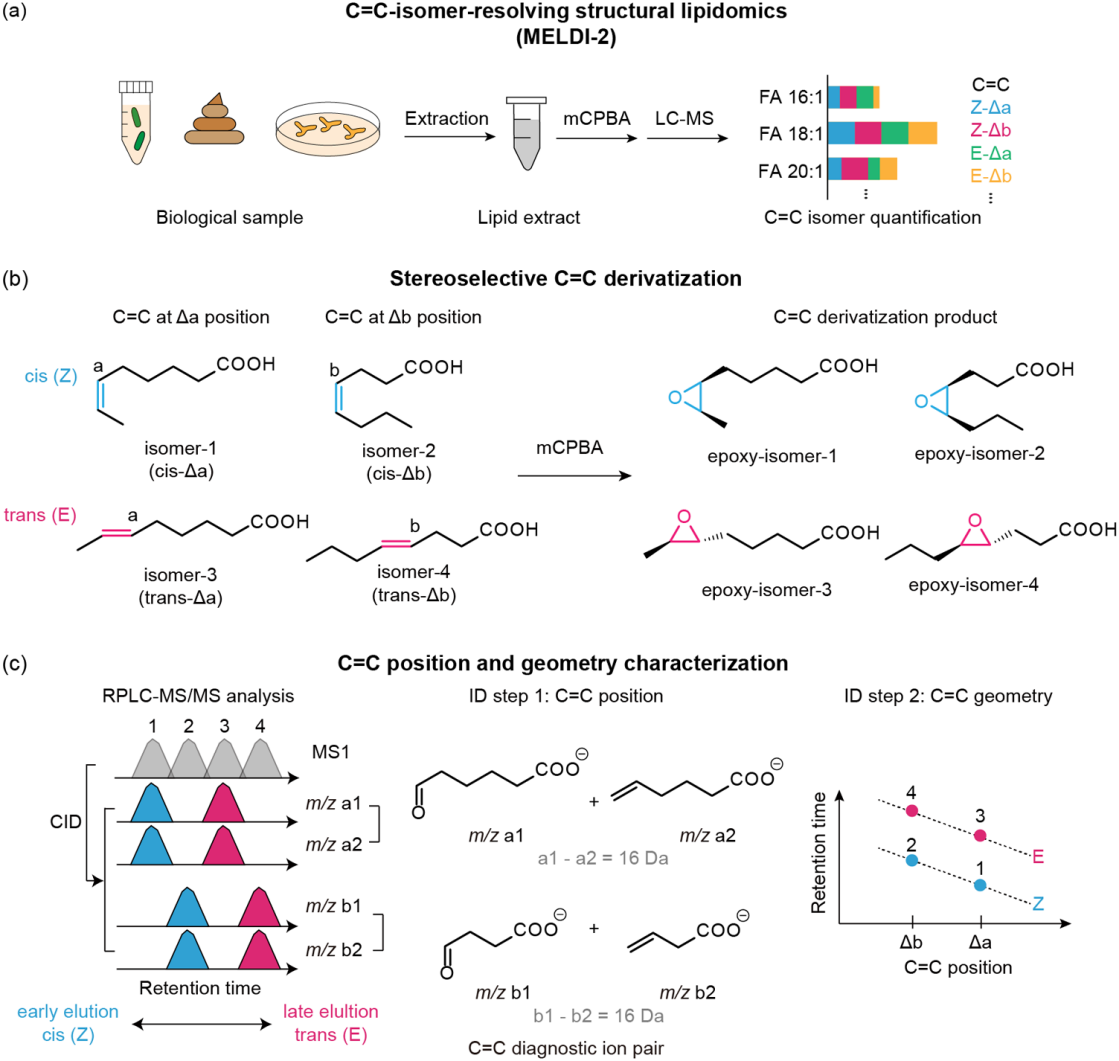
(a) Structural lipidomics workflow of MELDI-RPLC-MS/MS. (b) Stereoselective C=C derivatization by *m*CPBA. Unsaturated lipid isomers are derivatized by *m*CPBA, a rapid in-solution epoxidation process that transforms C=C bonds into epoxides with preserved *trans*/*cis* stereochemistry. (c) The derivatized isomers were analyzed through MELDI-RPLC-MS/MS and identified on the basis of their original C=C position and *trans*/*cis* geometry according to MS/MS diagnostic ion pairs and retention time sequences, respectively. After epoxidation derivatization and subsequent MS/MS using CID in negative ion mode, each C=C isomer yielded an aldehyde–alkene fragment pair indicative of the C=C position.

The working principle of MELDI-RPLC-MS/MS is depicted in Figure 1, which illustrates a hypothetical example in which four isomers differ in their C=C position, C=C geometry, or both. In the proposed method, *m*CPBA is used as a derivatization reagent to chemically modify lipid C=C bonds prior to MS analysis. The peracid *m*CPBA enables stereoselective epoxidation on the C=C bond to form an epoxide moiety (i.e., a three-membered ring with one oxygen heteroatom) with a preserved *trans*/*cis* geometry. The epoxide moiety is more fragile than C=C and thus enables direct C=C positional identification through conventional MS/MS (e.g., collision-induced dissociation (CID) in negative ion mode), a process where a lipid epoxide generates an aldehyde–alkene fragment pair specific to the C=C position (Figure 1b,c). However, the combined use of *m*CPBA epoxidation and MS/MS cannot easily distinguish between geometrical isomers because these isomers generate identical tandem mass spectra, as observed in our previous study.^36^

C=C geometry plays a determinant role in the hydrophobicity of an unsaturated lipid. Generally, the formation of the *cis*-C=C bond causes a fatty acyl chain to “bend”, thereby decreasing molecular hydrophobicity; by contrast, the formation of the *trans*-C=C bond leaves molecular hydrophobicity relatively unchanged. Taking advantage of this chemical property, the proposed method exploits RPLC analysis to provide an additional dimension of isomer-differentiating capability as well as MS/MS. As further explained by the hypothetical example in Figure 1b,c, the mixed isomers (epoxylipids) are chromatographically separated in RPLC-MS/MS analysis, with their tandem mass spectra continuously recorded over time. This analytical configuration enables the simultaneous identification of C=C geometry and C=C position in two steps. First, the C=C position is identified by peak alignment between the lipid and its C=C position-specific fragments (as measured through MS and MS/MS, respectively). Second, C=C geometry is identified on the basis of the retention time sequence. Given that a lipid with greater hydrophobicity is exposed to stronger retention in RPLC, a pair of *cis*/*trans* geometrical isomers results in two chromatographic peaks with the “*cis*-to-*trans*” order; that is, the *trans* isomer has a longer retention time than does the *cis* isomer.

### Method Validation with Lipid Isomer Standards

**Figure 2:**
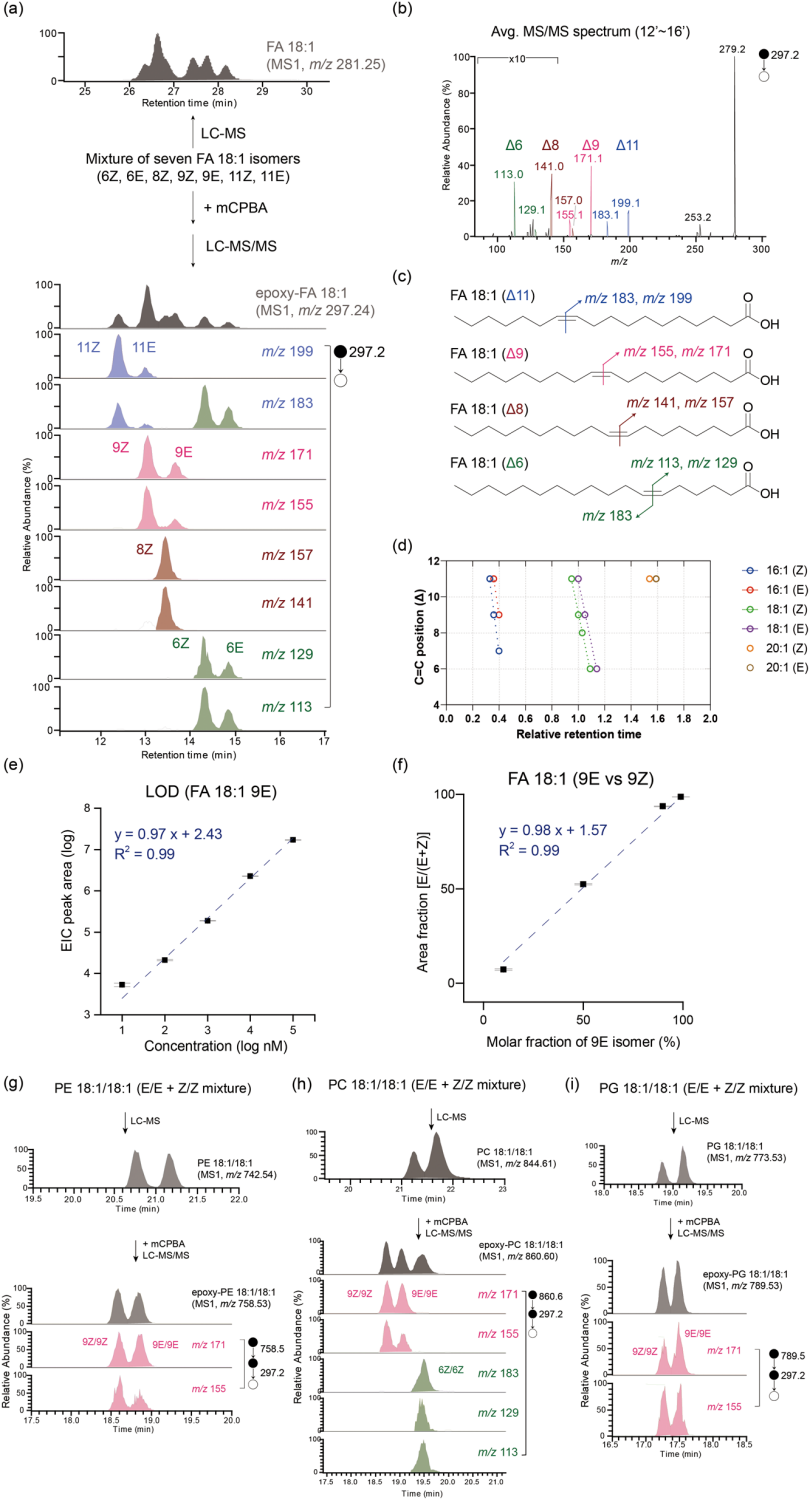
Developing and validating MELDI-RPLC-MS/MS with chemical standards of lipid isomers. (a–c) A standard mixture consisting of FA 18:1 isomers (unequal concentrations) was examined to demonstrate the analytical capability of the proposed method to identify C=C position and geometry. (a) EIC of nonderivatized and epoxidation-derivatized FA isomers; the same chromatographic parameters were used. (b) Representative tandem mass spectrum of epoxidation-derivatized isomers. Fragment pairs indicative of C=C positions are highlighted. (c) Fragmentation scheme. (d) Retention time index of the FA isomer standards. Linear fitting was performed on each series of C=C positional isomers with identical acyl chain lengths and *cis/trans* geometry. (e) LOD of the C=C-diagnostic ion pairs of FA 18:1 9E. (f) Calibration curve for *cis*/*trans*-isomer quantification of FA 18:1 (9Z versus 9E). In (e, f), each point and error bar represent the mean and standard deviation of technical triplicate values. In (e, f), “area” refers to the summed EIC peak area of the C=C-diagnostic ion pair in MELDI-RPLC-MS/MS analysis. (g–i) EICs of phospholipid epoxides and the diagnostic ions.

The C=C-resolving capability of the proposed method was validated systematically with authentic standards for two main lipid classes, namely, FA and glycerophospholipid. We applied the method to examine a mixture of 14 standards of monounsaturated FA (MUFA) that differed in terms of acyl chain length, C=C position, and C=C geometry, including five FA 16:1 isomers (Figure S1a–d), seven FA 18:1 isomers (Figure 2a–c), and two FA 20:1 isomers (Figure S1e,f). Four properties were used to identify C=C bonds. Taking the FA 18:1 isomer series as an example, we observed that (i) epoxidation derivatization generally decreased the hydrophobicity of all C=C isomers, resulting in an overall reduced retention time (Figure 2a); (ii) the C=C position was precisely indicated by Δ16-Da fragment pairs in MS/MS spectra (Figures 2b,c and S2) and their corresponding fragment chromatograms (Figure 2a) regardless of C=C geometry; (iii) as the C=C position moved farther from the carboxylic acid group (i.e., as the Δ number increased), the retention time decreased (Figure 2a); and (iv) for each pair of geometrical isomers, the *trans* and *cis* forms exhibited baseline separation and followed the “*cis*-to-*trans*” order in retention time, providing a foundation for the robust quantification of geometrical isomers (Figure 2a). These four observations were also valid for the FA 16:1 and 20:1 isomers (Figure S1). Furthermore, we discovered that for a given series of C=C positional isomers (e.g., FA 18:1 isomers with *trans*-C=C bonds at multiple positions), the retention time was linearly associated with the C=C position (Figure 2d); this mathematical relationship allows for the position of the C=C bonds in an unknown lipid to be precisely identified in the absence of its commercial or synthetic standard, which is crucial during large-scale screening of lipid isomers. Additionally, the limit of detection (LOD) for identifying C=C-diagnostic ions as assessed by FA 18:1 9E was approximately at the nanomolar (nM) level and thus enabled the identification of geometrical isomers of low abundance (Figure 2e).

To validate the quantification capability of the proposed method, we examined a mixture with FA 18:1 9Z and 9E isomers of varying ratios at a fixed total isomer concentration (described in the Experimental Section). The constructed calibration curve (Figure 2f) indicated a strong linear relationship (*R*^2^ = 0.99) between the C=C-diagnostic ion abundance (*y*-axis) and isomer composition (*x*-axis). The slope of the fitted calibration line (0.98) was also close to 1, implying that the ratio of C=C-diagnostic ion abundance served as a reliable estimator of the geometrical isomer composition. In addition to FA, the proposed method was also generally applicable for identifying the C=C position and C=C geometry in FA-constituting phosphoglycerolipids, including phosphoethanolamine (PE), phosphatidylcholine (PC), and phosphatidylglycerol (PG) (Figure 2g–i). Similar quantitative results were also obtained by analyzing a mixture of phospholipid geometric and positional isomers (Figure S3). In our previous study, we demonstrated the feasibility of measuring the compositions of positional isomers on the basis of C=C-diagnostic ion abundance.^36^ As demonstrated by the aforementioned findings, the proposed method can be used to effectively quantify the composition of both positional and geometric C=C isomers in a given unsaturated lipid species as a complement to traditional lipidomics results. Although C=C identification in polyunsaturated lipids can be challenging because of the presence of multiple derivatization products, in this study, we were able to identify the C=C position and C=C geometry through direct comparison of isomer spectra (as demonstrated by the FA 18:2 geometrical and positional isomers shown in Figure S4).

### Lipid C=C Isomer Analysis of Gut Bacterial Lipid Extracts

**Figure 3:**
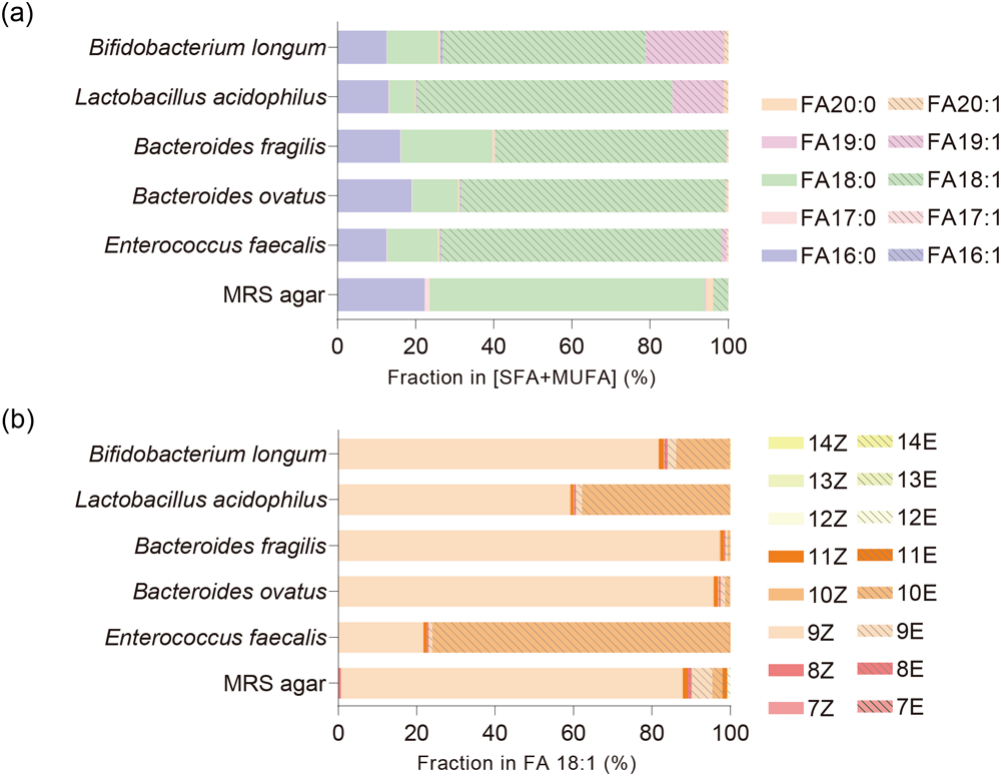
Saturated and unsaturated FA compositions in gut bacteria cultured in MRS. (a) Compositions of FAs (saturated FAs [SFAs] and MUFAs, characterized at the species level) in the lipid extracts from gut bacteria cultured on MRS agar. (b) C=C isomer composition of FA 18:1, with C=C position and geometry characterized.

**Figure 4:**
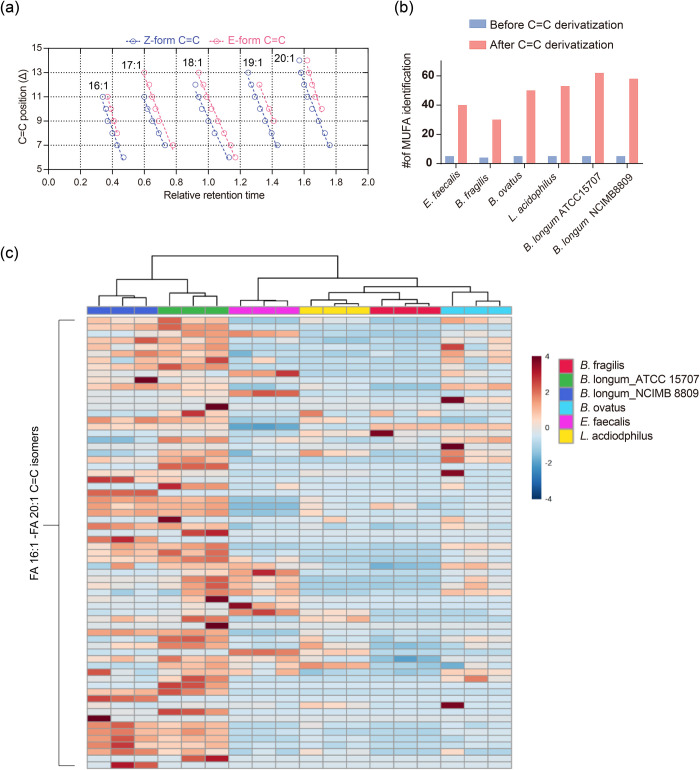
Application of MELDI-RPLC-MS/MS to structural lipidomics to investigate gut bacterial lipid isomerism. (a) Retention time index of the identified bacterial isomers. Linear fitting was performed on each series of C=C positional isomers with identical acyl chain lengths and *cis*/*trans* geometries. The data in (a) represent the means calculated from biological triplicates. (b) Identification number of MUFA before C=C derivatization (with the C=C position and geometry unresolved) and that of the MUFA C=C isomer species corresponding to unresolved MUFA species after C=C derivatization (with the C=C position and geometry resolved). (c) Clustering analysis of gut bacteria based on the FA C=C isomer profile.

To demonstrate the potential of the proposed method to analyze C=C isomers in biological samples, we analyzed lipid extracts from multiple common gut bacteria (Supplementary Table 1); the analytical workflow is depicted in Figure 1a. Specifically, we initiated the analysis by employing data-dependent acquisition (DDA) on samples without epoxidation. This process enabled us to obtain a list of unsaturated lipid candidates for epoxidation in each sample. The relative abundance of saturated FAs and MUFAs with carbon chains ranging from 16 to 20 in the gut bacterial extracts was determined on the basis of the MS^1^ EIC peak area, while the corresponding C=C positions and geometries remained unknown (Figure 3a). Subsequently, upon epoxidation, multiple peaks of C=C diagnostic ions were observed in the MS^2^ EICs (Figure S5), which enabled us to determine the C=C position and geometry and to calculate the relative abundance of the C=C isomers (Figures 3b and S6). In addition, the relative retention time of each peak of diagnostic ions was consistent with the standards (Figure 4a and Supplementary Table 2). Notably, more than 30 C=C isomers were identified in the gut bacterial extracts for the unsaturated FAs that we targeted, namely, FA 16:1–FA 20:1 (Figure 4b). These results not only suggested that our method substantially increased the identification number but also demonstrated the potential of gut bacteria to generate C=C isomers. By contrast, the number of phospholipid C=C isomers identified in the bacterial extracts was lower than that of unsaturated FAs. More specifically, the C=C diagnostic EICs exhibited one peak in most cases of unsaturated phospholipids, meaning that only one of the geometric isomers was observed (Figures S7 and S8).

To ensure the reproducibility of the proposed method, we cultured each gut bacterium in MRS in triplicate and obtained the corresponding C=C isomer profiles. A heatmap profile demonstrated the reproducibility of our method and showed that *Bifidobacterium longum* ATCC 15707 and *B. longum* NCIMB 8809 were clustered together, indicating similar C=C isomer compositions for the same gut bacteria species (Figures 4c and S9). Principle component analysis further indicated that gut bacteria species could be classified on the basis of their C=C isomer profiles (Figure S10). Notably, although the saturated and unsaturated FA compositions of these gut bacteria were similar (Figure 3a), the C=C isomer compositions differed (Figures 3b and S6). In addition to common unsaturated FAs such as FA 18:1 9Z, FA 18:1 11Z, and FA 20:1 11Z, we also identified other C=C isomers with relatively low abundance that have seldom been reported previously. Notably, we also found that FA 18:1 10E was a common unsaturated FA C=C isomer in the bacterial extracts with a relatively high abundance in *trans*-isomers (Figures 3b and S6). In the case of *Enterococcus faecalis*, the abundance was even higher than that of FA 18:1 9Z (Figure 3b), accounting for more than 50% of the abundance among all of the FA 18:1 C=C isomers, which indicated that FA 18:1 10E was the primary C=C isomer generated by gut bacteria. We also analyzed lipid extracts from the gut bacteria cultured in TSA medium and profiled the C=C isomer compositions. The results suggested that the C=C isomer compositions of the same gut bacteria species cultured in different media were notably different, indicating that the C=C isomer compositions of the gut bacteria were medium dependent (Figures S6 and S11), possibly because the different growth conditions affected the gut bacteria’s ability to generate C=C isomers in different ways or because some C=C isomers were absorbed from the medium involved.

### Integration with Isotope-Tracking Experiment to Determine the Origin of the C=C Isomer in Bacteria

**Figure 5:**
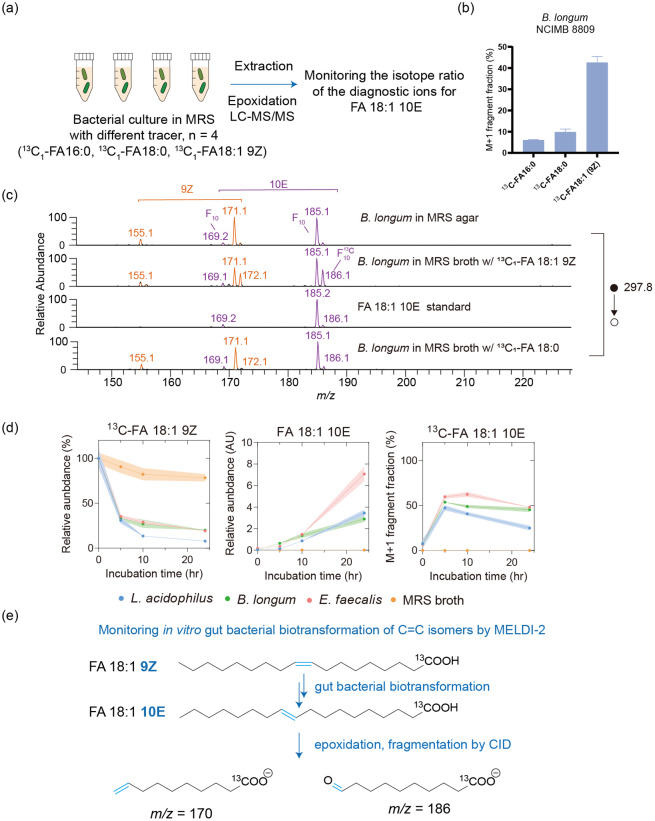
*In vitro* gut bacterial isomerization of FA 18:1 (9Z) into FA 18:1 (10E). (a) Experimental scheme. (b) M+1 fragment fraction of diagnostic ions for FA 18:1 10E in lipid extracts from *B. longum*. The M+1 fragment fraction was calculated by the formula (*A*_170_ + *A*_186_)/(*A*_169_ + *A*_185_ + *A*_170_ + *A*_186_). (c) MS/MS spectra of FA 18:1 epoxides in lipid extracts from *B. longum*. (d) Abundances of ^13^C_1_-FA 18:1 (9Z), FA 18:1 (10E), and ^13^C_1_-FA 18:1 (10E) in lipid extracts corresponding to multiple incubation times; the data represent the means calculated from biological triplicates. (e) Proposed putative isomerization mechanism and fragmentation mechanism.

The notably high abundance of FA 18:1 10E in gut bacterial lipid extracts captured our attention, motivating us to further investigate its biochemical origin. This observation may simply have resulted from bacterial uptake and the accumulation of medium contents, although FA 18:1 10E exhibited limited prevalence in media, as previously explained in quantifiable terms. To test whether FA 18:1 10E resulted from biotransformation activities by gut bacteria, we conducted an isotope-tracking experiment. This experiment involved culturing bacteria in the presence of an isotope-labeled compound as a biotransformation substrate and assessing whether the isotope-labeled atom was present in the target product, which in the present case was FA 18:1 10E. We tested three common FAs, namely, ^13^C_1_-FA 18:0, ^13^C_1_-FA 16:0, and ^13^C_1_-FA 18:1 9Z, which are generally recognized as precursors for downstream unsaturated lipid synthesis (Figure 5a). Following experiments with *B. longum*, we discovered that the supplementation of ^13^C_1_-FA 18:1 9Z, but not that of ^13^C_1_-FA 18:0 or ^13^C_1_-FA 16:0 (Figure 5b,c), specifically contributed to the isotope enrichment of FA 18:1 10E, as indicated by an elevated abundance of the ^13^C isotope of FA 18:1 9Z (referring to the 10E-specific diagnostic MS^2^ fragments, namely, *m*/*z* 186 and *m*/*z* 170, shown in Figure 5a) in association with a reduction of the precursor due to bacterial metabolism (Figure 5c). No isotope enrichment of other FA 18:1 isomers was observed, implying specificity of the 9Z-to-10E conversion. Similar results were obtained from the experiment with two other selected gut species, namely, *E. faecalis* and *Lactobacillus acidophilus* (Figure 5d). Overall, these findings suggest that the analyzed gut bacteria generated FA 18:1 10E through an unreported, putative isomeric biotransformation pathway with FA 18:1 9Z used as the substrate (Figure 5e); the detailed mechanism is currently under investigation.

**Figure 6:**
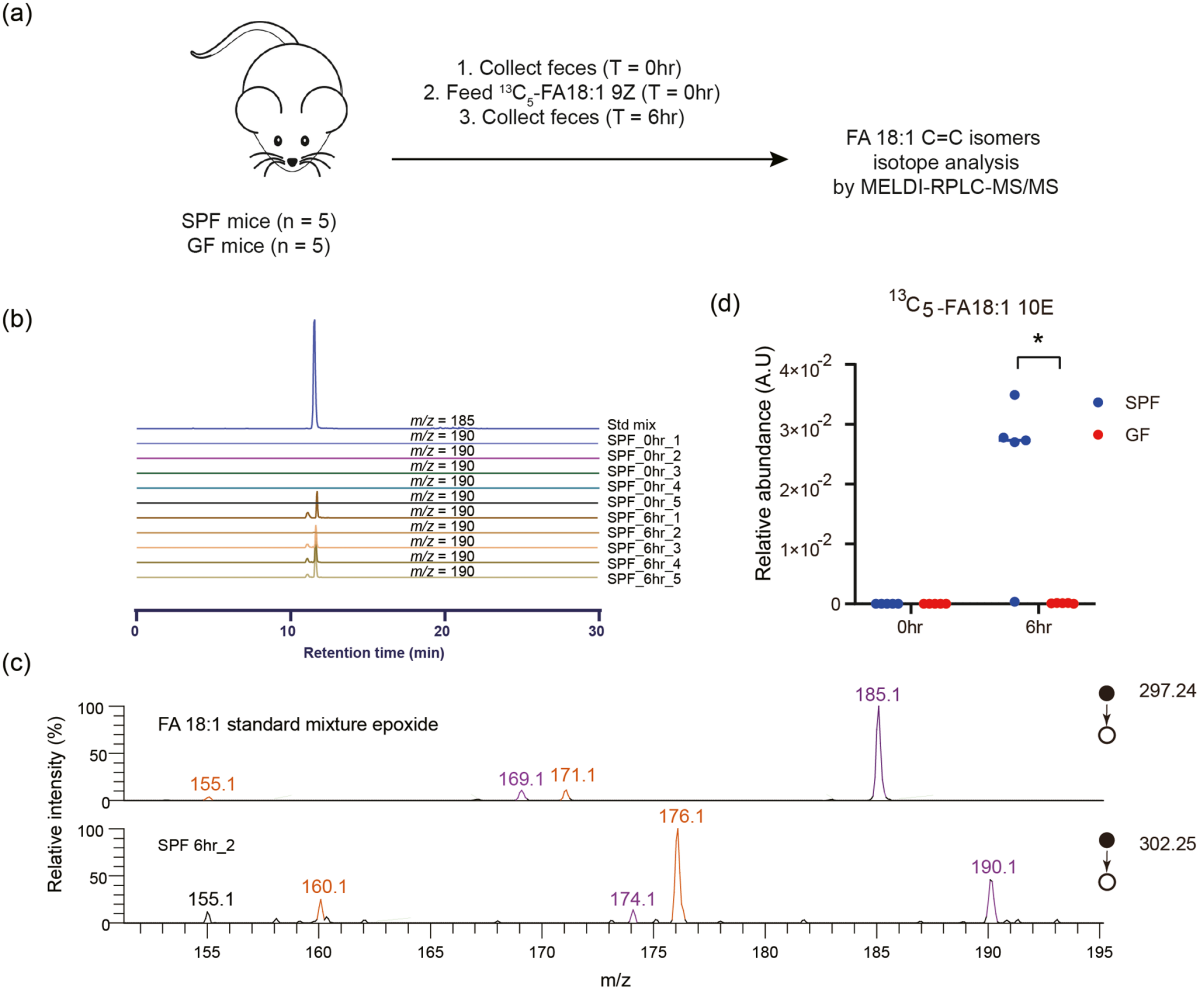
*In vivo* isotope tracking with ^13^C_5_-FA 18:1 9Z. (a) Experimental scheme. (b) MS^2^ EICs of the diagnostic ion for FA 18:1 10E and ^13^C_5_-FA 18:1 10E (*m*/*z* = 185: monoisotopic diagnostic ions for FA 18:1 10E, CID @ *m*/*z* = 297.24; *m*/*z* = 190: ^13^C_5_-labeled diagnostic ions, CID @ *m*/*z* = 302.25). Std mix: standard mixture of FA 18:1 9Z and FA 18:1 10E. SPF_ahr_b (“SPF” represents SPF mouse feces, “a” represents the time point when the feces was collected after isotope feeding started. “b” represents the biological replicates of mice (*n* = 5)): SPF mice feces. (a) Time point of feces collection after isotope feeding. (b) Number of mice (*n* = 5). (c) MS^2^ spectra of FA 18:1 epoxide. (d) Relative quantification of ^13^C_5_-FA 18:1 10E (normalized to the internal standard).

We have discovered that gut bacterial lipid extracts contain a diverse consortium of lipid C=C isomers. These observed bacterial C=C isomers are rarely discussed in mammalian lipidomics studies (possibly owing to analytical limitations), and this gap raises the question of whether the gut bacterial lipidome, as well as bacterial lipid metabolic activities, is an *in vitro* experimental defect and loses its relevance when gut bacteria are considered in the host’s gastrointestinal tract. We thus attempted to address this challenge by further applying an isotope-tracking experiment similar to animal experiments. In a pilot study, we utilized GF mice for comparison with SPF mice to determine whether the presence of gut bacteria played a role in the FA 18:1 9Z-to-10E conversion (Figure 6a). Six hours after oral administration of isotope-labeled FA 18:1 9Z as a metabolic tracer, we performed target quantification of fecal FA 18:1 C=C isomers and found substantially higher amounts of the isotope-labeled 10E isomer in SPF mice than in GF mice, in which the corresponding amounts were undetectable (Figures 6b,c and S12). In addition, the amount of unlabeled FA 18:1 10E was higher in the SPF mice than in the GF mice (Figure S13). Interestingly, we also observed a higher amount of tracer retained in the SPF mouse feces (Figure S13), implying that the metabolism of the 9Z isotope tracer may have been faster in the GF mice and yet did not reflect the bacteria-independent production of the 10E isomer. Overall, our preliminary results suggest that the presence of bacteria may be essential to producing FA 18:1 10E in the gut and that bacterial biotransformation of FA 18:1 9Z to its 10E isomer may occur in the gut. Although this conclusion requires further validation through experiments involving bacterial genetics and gnotobiology analyses to identify responsible bacterial species and enzymes, our structural lipidomics workflow is useful for elucidating the gut bacterial metabolic pathway by offering precise lipid C=C isomer quantification.

## Conclusion

This study combined MELDI with RPLC-MS/MS as a user-friendly method for identifying and quantifying unsaturated lipid C=C isomers. We validated this proposed method by analyzing an artificial standard mixture and gut bacterial lipid extracts. Furthermore, we successfully identified a series of positional and geometric unsaturated C=C isomers and profiled the isomer composition of the gut bacterial lipid extracts with the C=C position and C=C geometry being characterized. Notably, the *in vivo* and *in vitro* isotope-tracking experiments suggested a previously unreported gut bacterial biotransformation pathway where FA 18:1 9Z was converted into FA 18:1 10E. Given the negative effect of *trans*-unsaturated FAs on human health, this pathway could constitute a key interaction between gut bacteria and host.

## Supporting Information Available

Supplementary table (XLSX)
EpoxyFinder software (ZIP)
Supplementary methods and figures (PDF)
