# Supplementary material for: Mapping Lipid C=C Isomer Profiles of Human Gut Bacteria through a Novel Structural Lipidomics Workflow Assisted by Chemical Epoxidation
Source: Anal Chem. 2024 Oct 22;96(44):17526–36. doi: 10.1021/acs.analchem.4c02697 (PMC11541895; doi:10.1021/acs.analchem.4c02697)

EpoxyFinder User Manual

■ Install EpoxyFinder

1. If MATLAB runtime R2018a (ver 9.4) has already been installed, just double click EpoxyFinder.exe
2. If no MATLAB runtime R2018a (ver 9.4) be installed, double click AppInstaller_EpoxyFinder.exe to install Matlab runtime.
   a. Select directory for installing matlab runtime

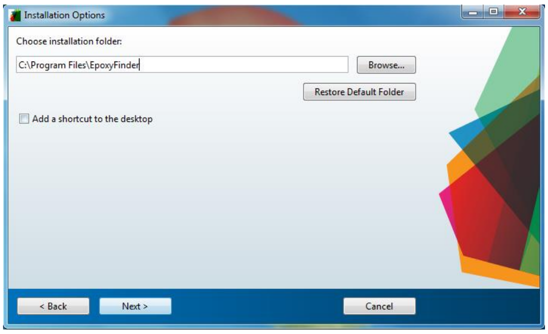

   b. Click Next >

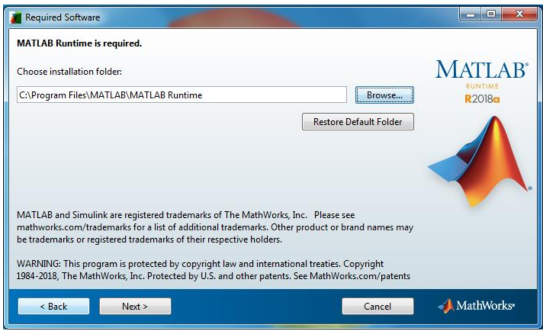

   c. Agree the license and click Next >

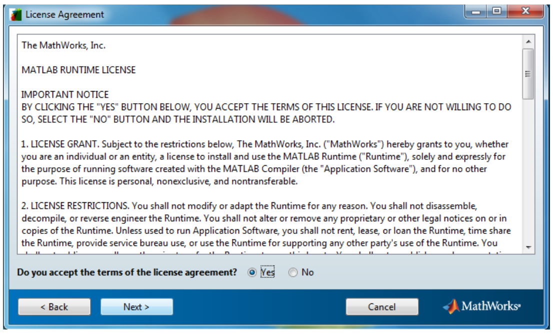

3. After the Matlab runtime being installed, you can open the program by double click the EpoxyFinder.exe.

■ Prepare lipid list for EpoxyFinder

The lipid list is required to specify lipids for further C=C position analysis. The lipid list should contain four columns, including LipidIon, Class, ClacMz, and GroupArea. The header of the columns should be included (case sensitive), and the file should be saved as .xlsx format.

1. LipidIon
   This column is used to specify the number of double bond in the lipid. The number of carbon and the number of double bond should be separate by colon (:). For example, fatty acid with 15 carbons and one double bond should be typed as FA(15:1). If there are two acyl chains, separate the two acyl chains by a slash (/).
2. Class
   If the lipid is a fatty acid, type FA (case sensitive). There is no limitation for other classes of lipids.
3. CalcMZ
   The accurate mass for the lipids. Notice that input the mass of [lipid – H].
4. GroupArea
   The GroupArea column is used to exclude lipids with low abundance. If you do not want to use this function, just type 0.

LipidIon	Class	CalcMz	GroupArea
FA(14:0)-H	FA	227.2017	5441570.665
FA(15:1)-H	FA	239.2017	411869.724
FA(16:2)-H	FA	251.2017	1911878.812
LPA(16:1)-H	LPA	407.2204	90660.702
LPA(16:0)-H	LPA	409.2361	4474434.894
LPA(18:2)-H	LPA	433.2361	167044.826
PA(16:0/14:0)-H	PA	619.4344	9.20E+07
PA(16:0/16:1)-H	PA	645.4501	87990.74
PA(15:0/18:1)-H	PA	659.4657	4756761.89
PA(16:0/17:1)-H	PA	659.4657	2989623.984

■ Using EpoxyFinder

1. Select the lipid list by clicking the Browse button. Notice that that lipid list file should be in the format of .xlsx. Besides, four column headings should be included, including LipidIon, Class, CalcMz, and GroupArea (case sensitive).

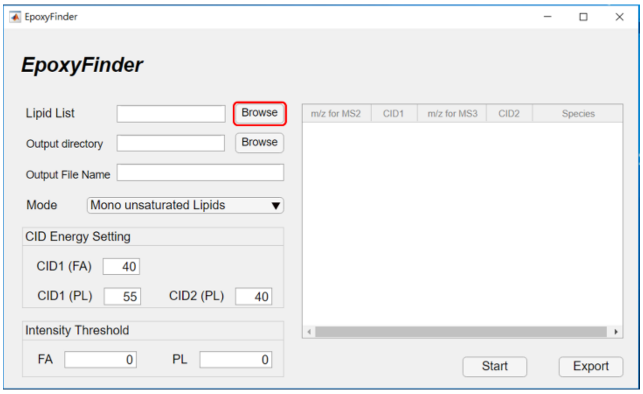

2. Select the directory and type the filename for the output file. The default directory is the same as the lipid list file directory.

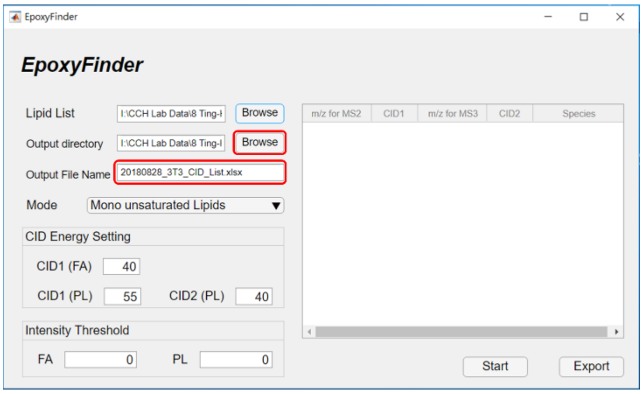

3. Select the mode for EpoxyFinder. The mono unsaturated lipids mode targets only the lipids with one double bond. In contrast, the poly unsaturated lipids mode targets lipids with more than one double bonds.

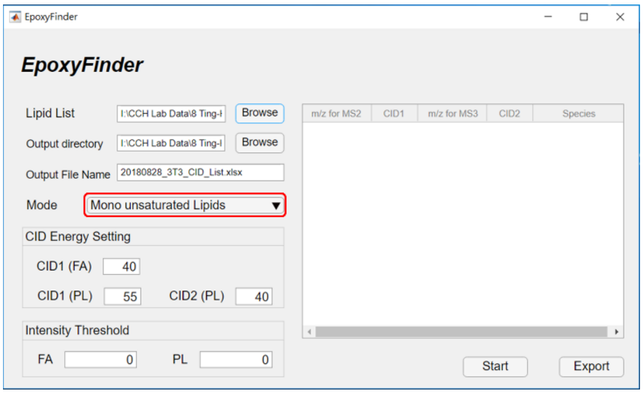

4. In the CID energy-setting dialog, you can set the collision energy for the MS^2 and MS^3 analysis. FA is the abbreviation for fatty acids, while PL is for phospholipids.

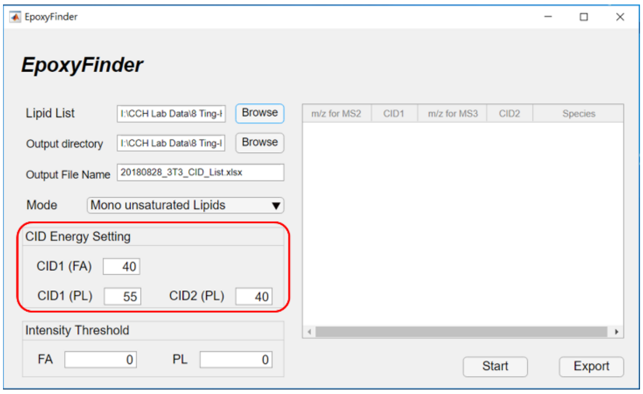

5. In the intensity threshold dialog, you can exclude unsaturated lipids with low abundance. If you do not want to filter base on the intensity, just input 0.

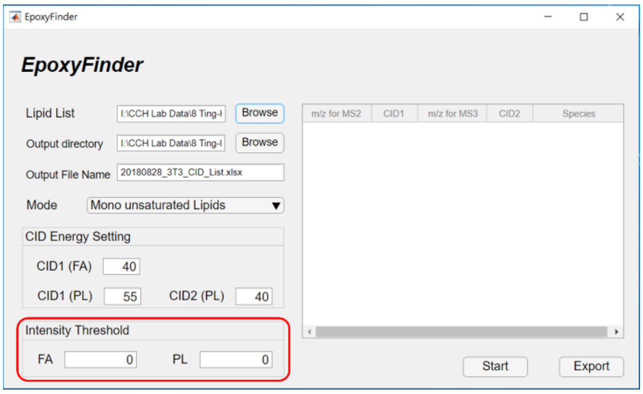

6. After input all the parameters, just click Start. It may take a while for the EpoxyFinder to create the CID list. The list will be shown in the table on the right side. The contents in the table is editable by double clicking.

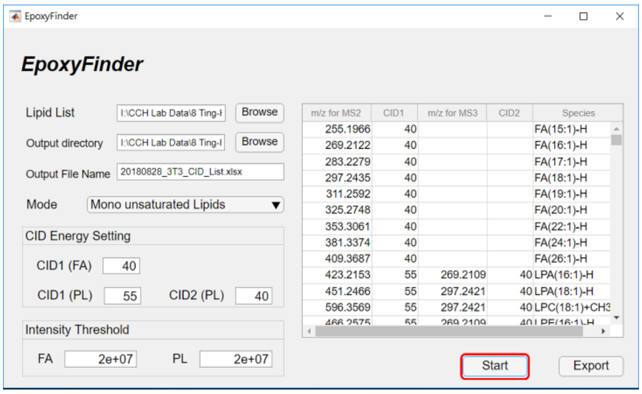

7. Click Export to save the list as a excel file for further analysis.

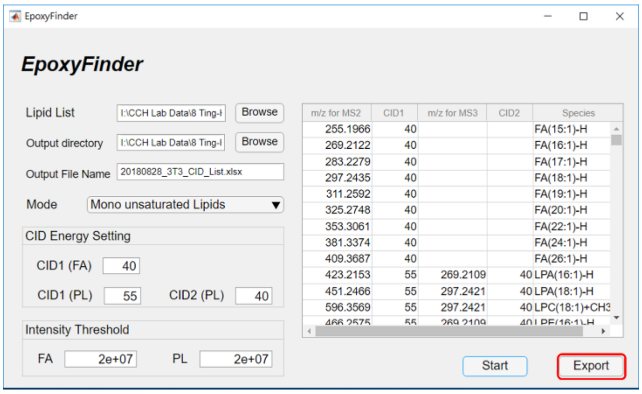

Supplement: Supplementary file 2 — ac4c02697_si_002.zip [file ac4c02697_si_002.zip › EpoxyFinder User Manual.pdf]
