# Supplementary material for: Mapping Lipid C=C Isomer Profiles of Human Gut Bacteria through a Novel Structural Lipidomics Workflow Assisted by Chemical Epoxidation
Source: Anal Chem. 2024 Oct 22;96(44):17526–36. doi: 10.1021/acs.analchem.4c02697 (PMC11541895; doi:10.1021/acs.analchem.4c02697)
Supplement: Supplementary file 3 — ac4c02697_si_003.pdf [file ac4c02697_si_003.pdf]

Supplementary information for

Mapping lipid C=C isomer profiles of human gut bacteria through a novel structural lipidomics workflow assisted by chemical epoxidation

Kai-Li Chen[†], Ting-Hao Kuo[†], Cheng-Chih Hsu[*†,‡].

[†] Department of Chemistry, National Taiwan University, Taipei 10617, Taiwan

[‡] Leeuwenhoek Laboratories, Co. Ltd. Taipei, Taiwan

Contact information

Cheng-Chih Hsu*

Email: ccrhsu@ntu.edu.tw

Phone: +886-2-33663844

Contents of supplementary information

1. **Supplementary materials and methods**

Untargeted LC-MS/MS lipid analysis of gut bacterial lipid extracts.

Predicted potential targeted epoxy-lipid precursor ion lists for LC-MS-tPRM analysis.

Bacteria culture by TSA agar

The calibration curve for relative quantification of PG 18:1/18:1 (9Z/9Z) and (9E/9E) isomers.

2. **Supplementary figures**

Figure. S1. Monounsaturated fatty acid mixture analysis.

Figure. S2. The individual MS^2 spectrum of FA 18:1 epoxides.

Figure. S3. Calibration curve of the phospholipid standards.

Figure. S4. Polyunsaturated fatty acid standards analysis.

Figure. S5. MS^2 EICs of FA16:1-FA20:1 epoxide in the lipid extracts from *B. longum* NCIMB8809.

Figure. S6. FA16:1-FA20:1 C=C isomer profiling of lipid extracts from the gut bacteria cultured in MRS agar.

Figure. S7. The EIC and MS^3 spectra of the unsaturated phospholipid epoxide in the bacterial extracts from *B. longum* DSM20090 cultured in TSA.

Figure. S8. Phospholipids C=C positional isomer profiling of the lipid extracts from the gut bacteria cultured in TSA agar.

Figure. S9. Heatmap of the C=C isomers for 6 gut bacteria.

Figure. S10. PCA analysis of gut bacterial lipid extracts.

Figure. S11. FA 16:1-FA 20:1 C=C isomer profiling of the lipid extracts from the gut bacteria cultured in TSA agar.

Figure. S12. *In vivo* isotope tracking with germ-free mice.

Figure. S13. Quantification of FA 18:1 10E & $^{13}C_5$-FA18:1 9Z in the SPF mice feces and GF mice feces

Supplementary method

Untargeted LC-MS/MS lipid analysis of gut bacterial lipid extracts.

For untargeted lipid analysis of lipid extracts from gut bacteria, a top-10 data-dependent acquisition (DDA) method was established. The scanning cycle of top-10 DDA consists one full FT-MS scanning with mass range of m/z 200-2000 and spectral resolution of 120,000, followed by 10 DDA IT-MS/MS scanning events. The minimal threshold to trigger MS/MS was 0.0. The dynamic exclusion was enabled after 2 repeat counts with 3 seconds of each repeat duration, an exclusion list size of 200, and an exclusion duration of 20 seconds. The exclusion mass width is set of +/- 10 ppm relative to the reference mass. MS/MS spectra were acquired via ion activation type of CID with default charge state of 1, isolation width of *m/z* 2.0, normalized collision energy (NCE) of 35.0, activation Q of 0.250, and activation time of 10.00 ms. The maximum injection time for both full FT-MS and IT-MS^n was set at 500 ms with auto-gain-control (AGC) of 1.00e+6 for full FT-MS scan and 3.00e+4 for IT MS/MS. The data was collected with Xcalibur 3.0 (Thermo Scientific).

Predicted potential targeted epoxy-lipid precursor ion lists for LC-MS-tPRM analysis. The LC-MS lipidomics data were processed via LipidSearch™ (version 4.0, Thermo Scientific) in the SearchType "Product" and the ExpType "LC-MS". The settings were optimized for lipid identification by high-resolution MS^1 precursors m/z and MS/MS fragments. Product ions with ion intensity above the 1.0% threshold were searched and identified with a precursor tolerance of 10.0 ppm and product tolerance of 0.5 Da. Both FA and all GPL classes were targeted for identification, and their negative-polarity ion adducts were chosen, including -H^+ and + $CH3COO^-$ (for PC) adducts. Then the search results were processed in the alignment mode to generate the report, containing all the identified lipids. The alignment result was then imported into an Excel file and processed through EpoxyFinder, a lab-built MATLAB-based software, to create a tPRM precursor ion list of the targeted mono-epoxidized unsaturated lipids for subsequent C=C isomer analysis. In general, this list included MS/MS and MS/MS/MS precursor ion m/z parameters of mono-epoxidized unsaturated lipids and the corresponding NCE.

The phospholipid standards mixture analysis. The phospholipid standard mixture was prepared by mixing PG 18:1/18:1 (9Z/9Z), PG 18:1/18:1(9E/9E), PC 18:1/18:1(6Z/6Z), PC 18:1/18:1(9Z/9Z), PC 18:1/18:1(9E/9E), PE 18:1/18:1(9E/9E), and PE 18:1/18:1(9Z/9Z) with the final concentration of each isomer at 1 μM. The mixture was then derivatized with excess mCPBA (50 mM) at 50°C for 1 hour and then analyzed by LC-MS. For the targeted unsaturated phospholipid C=C isomers analysis, a targeted MS^3 method was established. The scanning cycle consists of one full FT-MS scanning with a mass range of 200-1000 and spectral resolution of 60000, followed by 3 targeted IT-MS^3 scanning events. The MS^3 spectra of 3 targeted monoepoxy-precursor ions, PG18:1/18:1 (*m/z* = 789.5), PC18:1/18:1 (*m/z* = 860.6), and PE18:1/18:1 (*m/z* = 758.5) were acquired via first ion activation type of CID followed by second CID targeted at *m/z* = 297.2. The isolation width of the parent

ion was *m/z* 3.0 for MS/MS and *m/z* 5.0 for MS^3. The NCE value was set at 55.0 for MS/MS and 40.0 for MS^3. Data was collected with Xcalibur 3.0 (Thermo).

The calibration curve for relative quantification of PG 18:1/18:1 (9Z/9Z) and (9E/9E) isomers. The total concentration of PG 18:1/18:1 was kept at 10 μM with the molar ratio varied ([9E/9E]/[9Z/9Z] = 99/1, 90/10, 50/50, 10/90, 1/99). Each mixture was derivatized with excess mCPBA (20 mM) at 50℃ for 1 hour and then analyzed by LC-MS, where the mono-epoxide of PG 18:1/18:1 (*m/z* = 789.5) was targeted. The calibration curve was constructed by plotting the molar fraction of 9E isomers ([9E/9E]/([9E/9E]+[9Z/9Z])%) against the fractions of the summed extracted ion chromatographic(XIC) area of diagnostic ions ($A_{9E/9E}/(A_{9E/9E} + A_{9Z/9Z})$%), where $A_{9E/9E} = A_{trans,\ m/z = 155} + A_{trans,\ m/z = 171}$, and $A_{9Z/9Z} = A_{cis,\ m/z = 155} + A_{cis,\ m/z = 171}$, both obtained from the MS^3 channel at m/z 789.5, m/z 297.2.

Polyunsaturated fatty acid standards analysis.

The polyunsaturated fatty acid standards (FA18:2 9Z,12Z; FA18:2 9E, 12E; FA18:2 9Z,11Z; FA18:2 9Z, 11E) were used in this study. Each standard solution was prepared in MeOH at 100 μM. The standard solution was epoxidated by the addition of an equal volume of *m*CPBA (20 mM) at 50 °C for 1 hour and then analyzed by RPLC-MS/MS. For the targeted unsaturated polyunsaturated fatty acid analysis, a targeted MS^2 method was established. The scanning cycle consists of one full FT-MS scanning with a mass range of 200-1000 and spectral resolution of 60000, followed by 2 targeted IT-MS^2 scanning events. The MS/MS spectra of 2 targeted epoxy-precursor ions, mono-epoxide of FA 18:2 (*m/z* = 295.23) and di-epoxide of FA18:2 (*m/z* = 311.22), were acquired via ion activation type of CID, isolation width of *m/z* 2.0, normalized collision energy (NCE) of 40.0, activation Q of 0.250, and activation time of 10.00 ms. The maximum injection time for both full FT-MS and IT-MS^n was set at 500 ms with auto-gain-control (AGC) of 1.00e+6 for full FT-MS scan and 3.00e+4 for IT-MS/MS. The data was collected with Xcalibur 3.0 (Thermo Scientific).

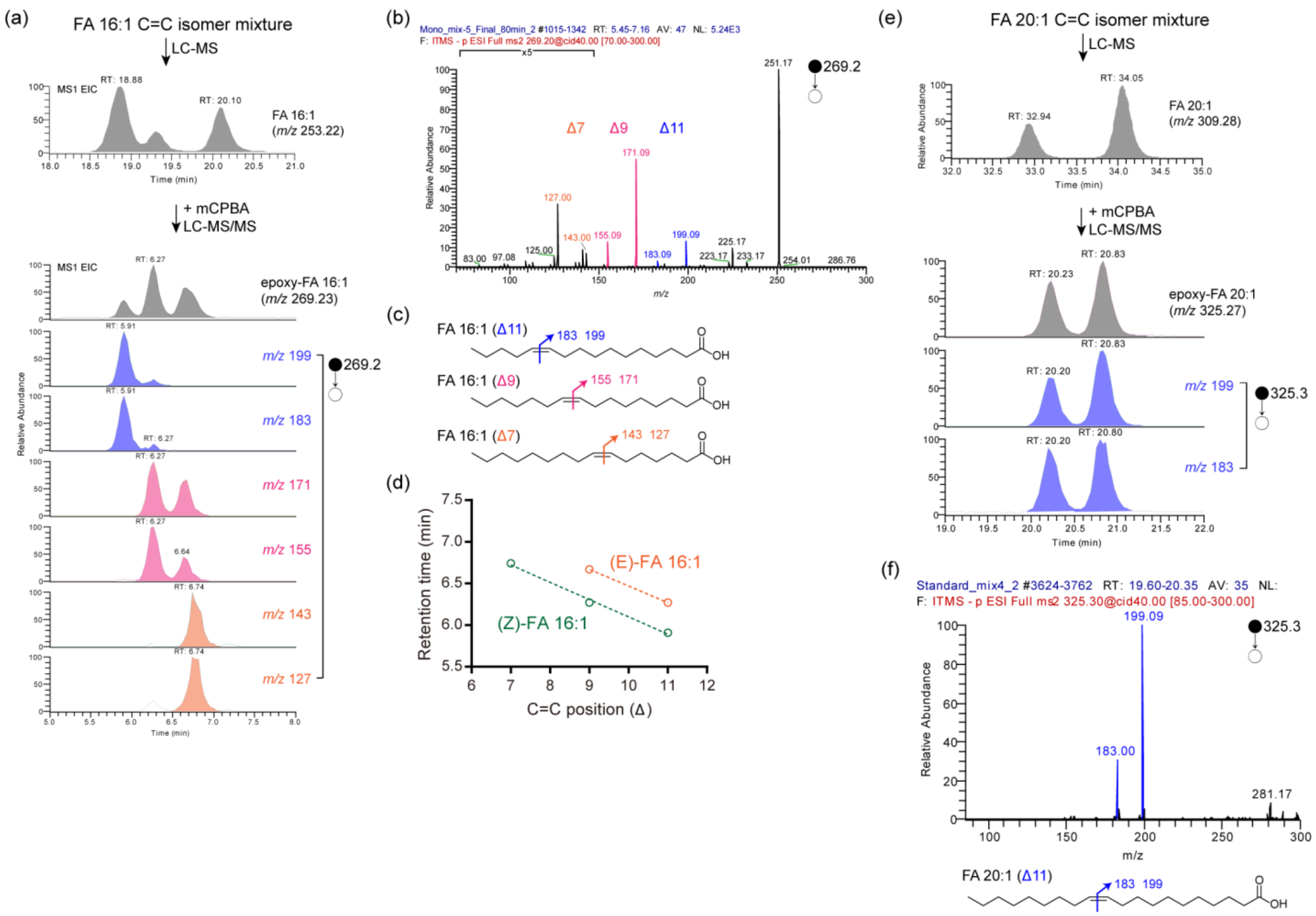

Figure. S1 Monounsaturated fatty acid mixture analysis. (a) MS[1] EIC of FA16:1 (*m/z* = 253.22), epoxide (269.23) and MS[2] EICs of C=C diagnostic ions (*m/z* = 127, 143, 155, 171, 183, 199). (b) MS[2] spectra of FA16:1 epoxide (c) Cleavage site of FA16:1 and *m/z* of diagnostic ions. (d) Retention time FA16:1 E/Z epoxide. (e) MS[1] EIC of FA20:1 (*m/z* = 309.28), epoxide (*m/z* = 325.27) and MS[2] EICs of C=C diagnostic ions (*m/z* = 183, 199) (f) MS[2] spectra of FA20:1 epoxide. The concentration of each isomer ranged from 0.1 μM to 10μM

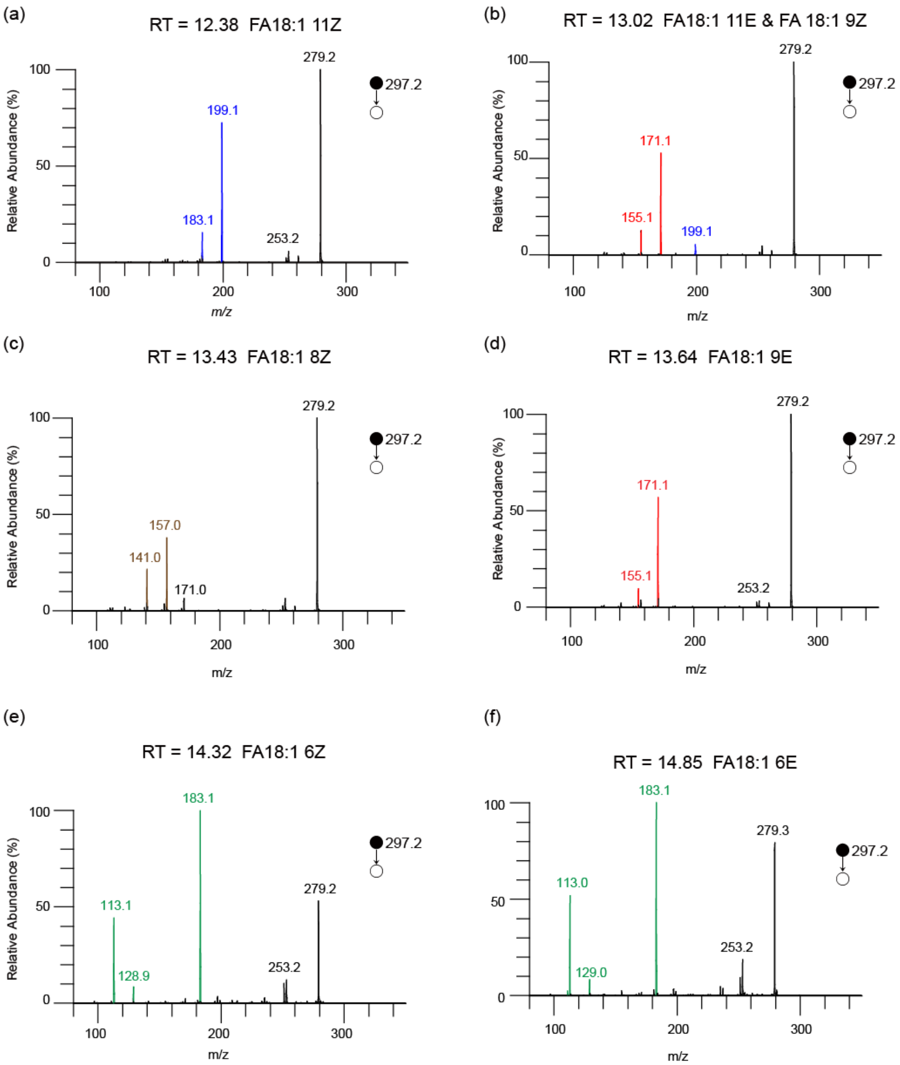

Figure. S2 The individual MS^2 spectrum of FA 18:1 epoxides.

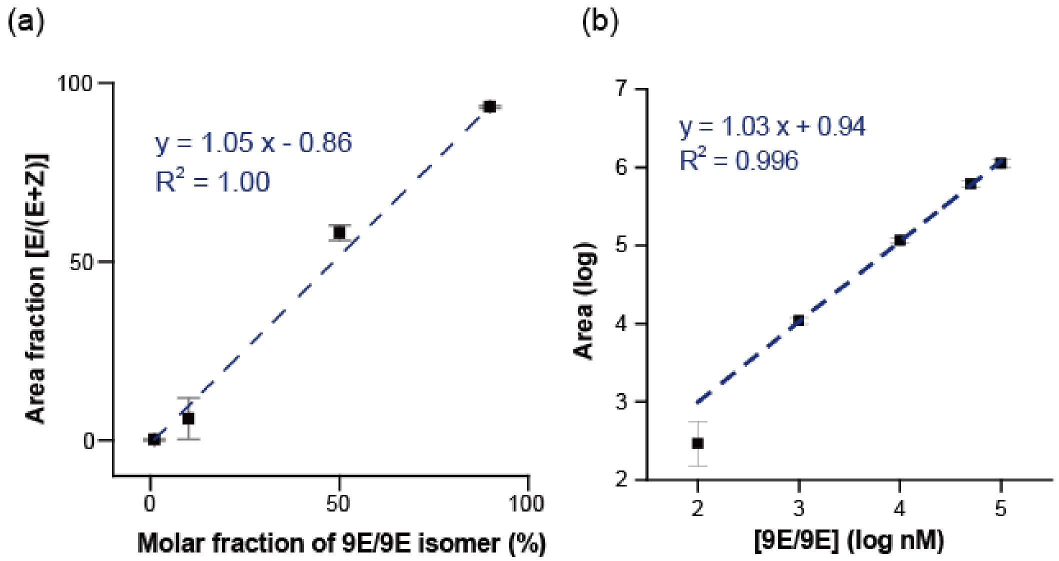

Figure. S3 Calibration curve of the phospholipid standards. (a) PG 18:1/18:1 (Δ9) (E-Z mixture) (b) PG 18:1/18:1 (9E/9E).

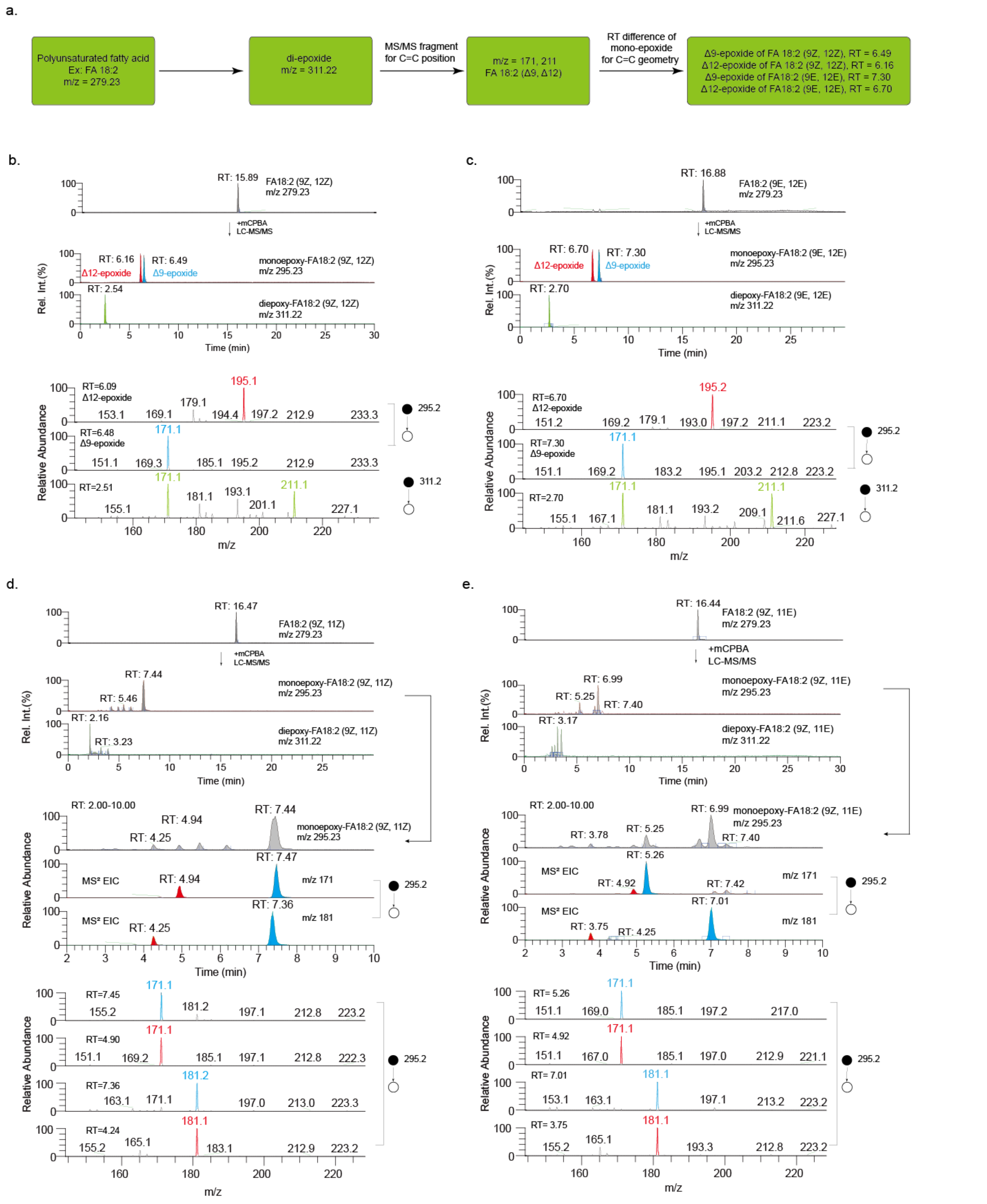

Figure. S4 Polyunsaturated fatty acid standards analysis. (a.) EICs of FA 18:2(9Z, 12Z), monoepoxy-

FA18:2(9Z, 12Z) and diepoxy-FA 18:2(9Z, 12Z) and MS^2 spectra of epoxide. (b.) EICs of FA 18:2(9E, 12E), monoepoxy-FA 18:2(9E, 12E) and diepoxy-FA 18:2(9E, 12E) and MS^2 spectra of epoxide. (c.) EICs of FA 18:2 (9Z, 11Z), monoepoxy-FA 18:2 (9Z, 11Z) and diepoxy-FA 18:2(9Z, 11Z) and MS^2 spectra of epoxide. (d.) EICs of FA 18:2 (9Z, 11E), monoepoxy-FA 18:2 (9Z, 11E) and diepoxy-FA 18:2(9Z, 11E) and MS^2 spectra of epoxide and MS^2 spectra of epoxide.

B. longum NCIMB 8809 FA16:1 MS² EICs

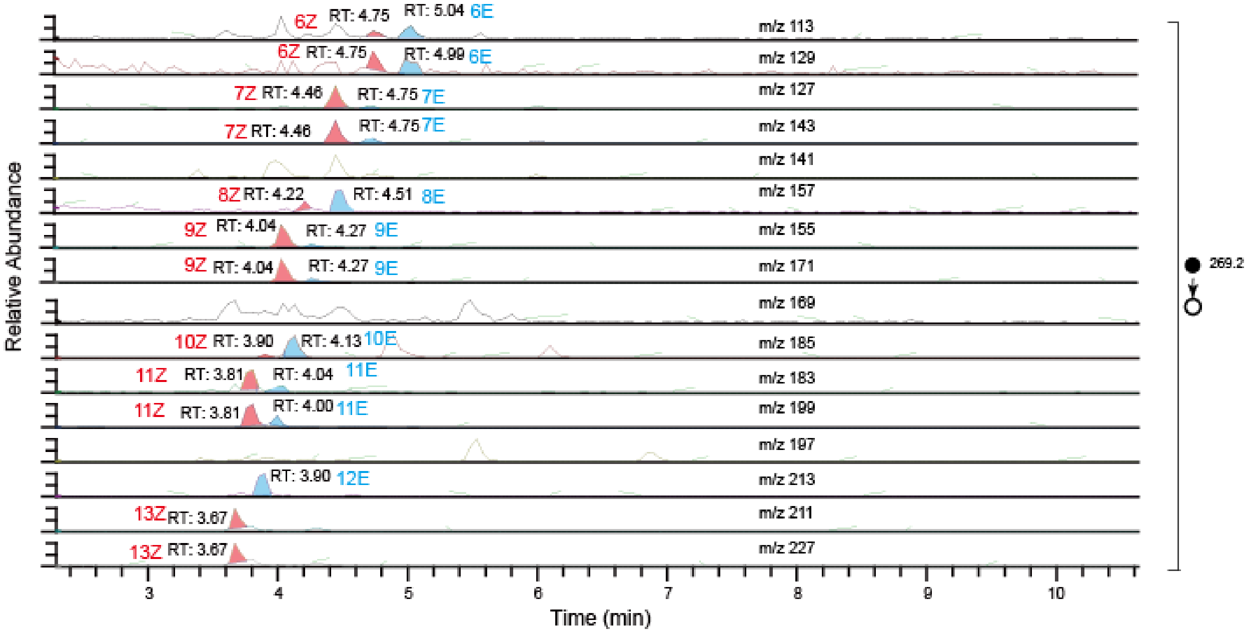

B. longum NCIMB 8809 FA17:1 MS² EICs

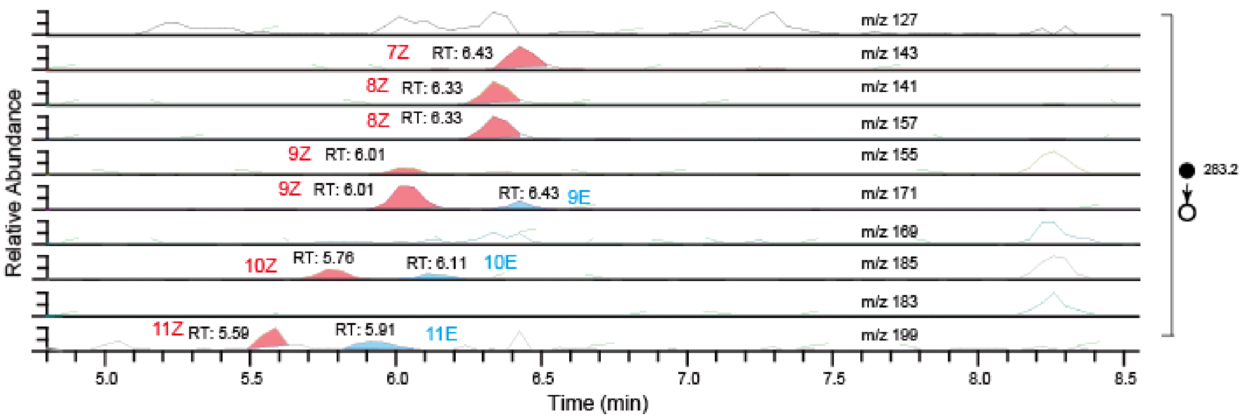

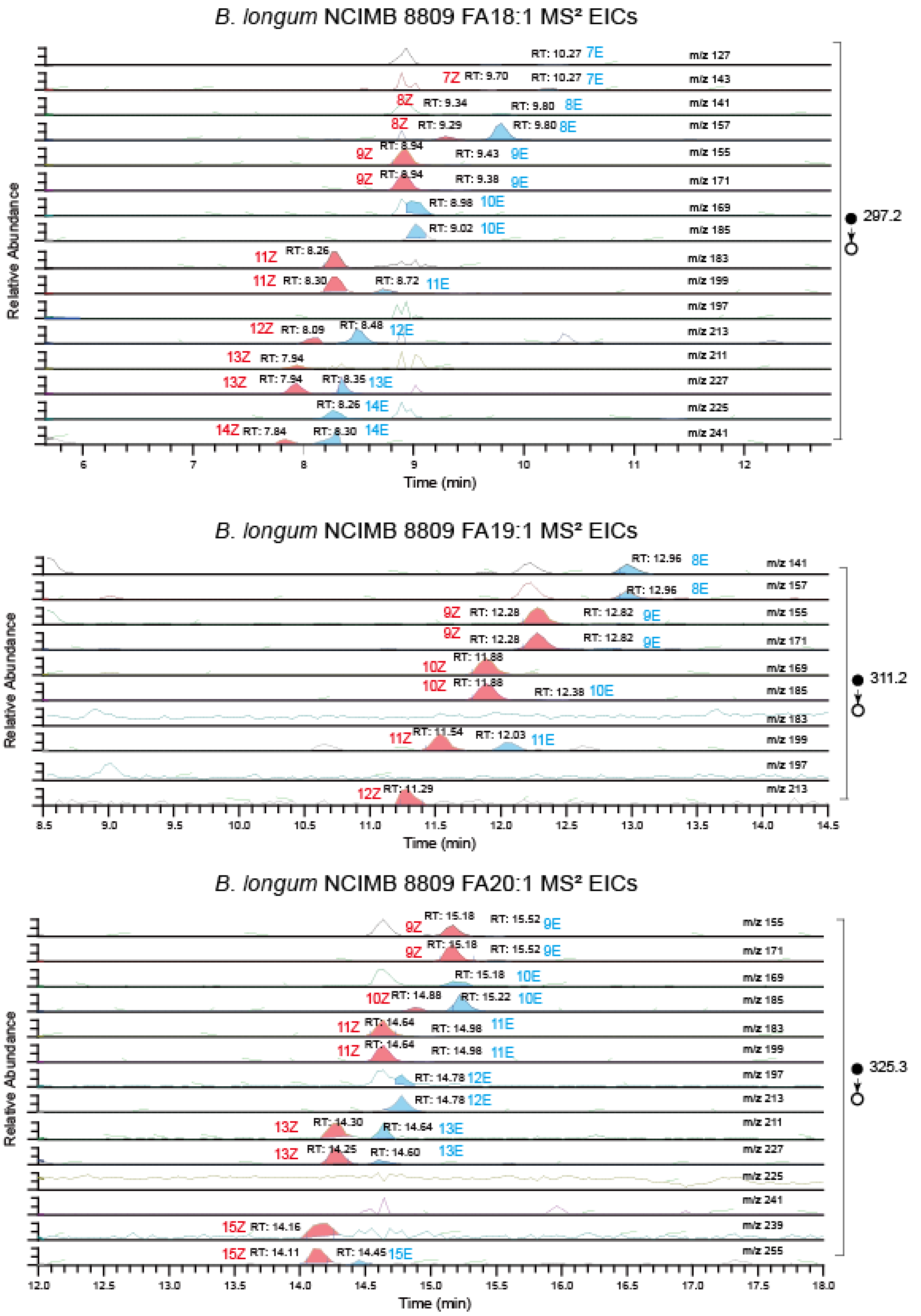

Figure. S5 MS[2] EICs of FA 16:1-FA 20:1 epoxide in the lipid extracts from *B. longum* NCIMB 8809.

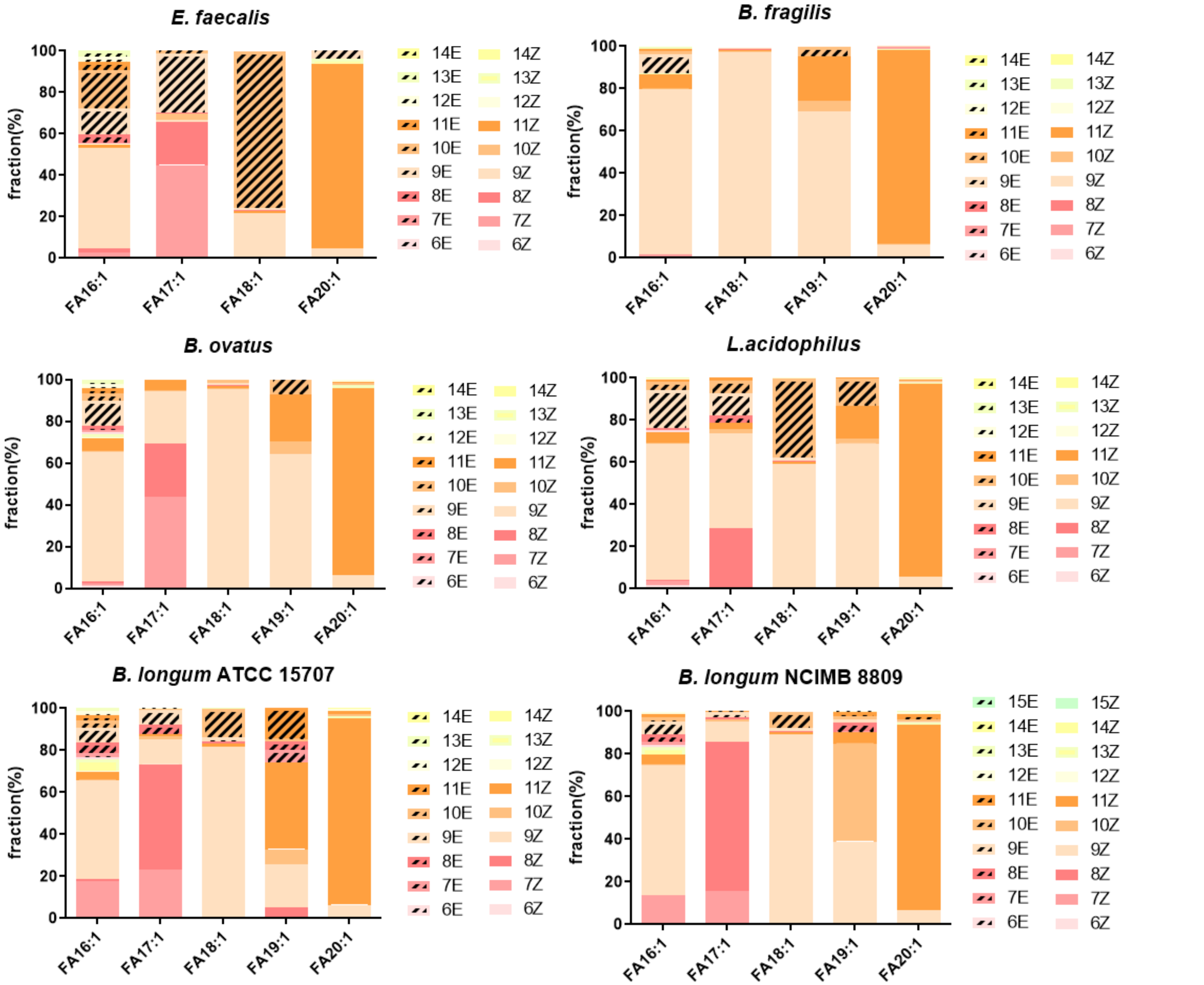

Figure. S6 FA 16:1-FA 20:1 C=C isomer profiling of lipid extracts from the gut bacteria cultured in MRS agar. (Each data is the average of triplicate samples)

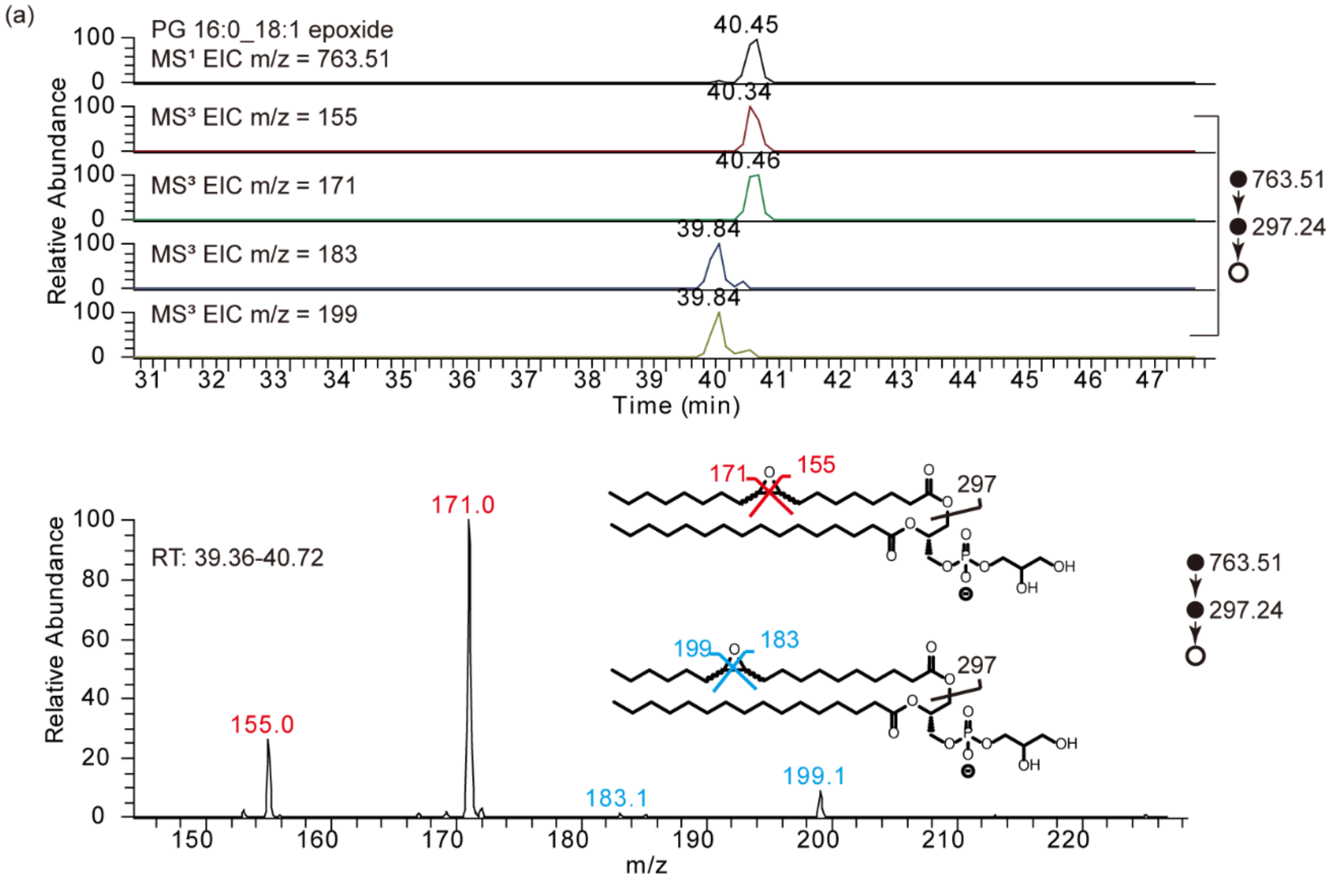
(a)
PG 16:0_18:1 epoxide
MS¹ EIC m/z = 763.51
40.45
MS³ EIC m/z = 155
40.34
MS³ EIC m/z = 171
40.46
MS³ EIC m/z = 183
39.84
MS³ EIC m/z = 199
39.84
763.51
297.24
Relative Abundance
Time (min)
RT: 39.36-40.72
171.0
155.0
183.1
199.1
171
155
297
199
183
OH
m/z

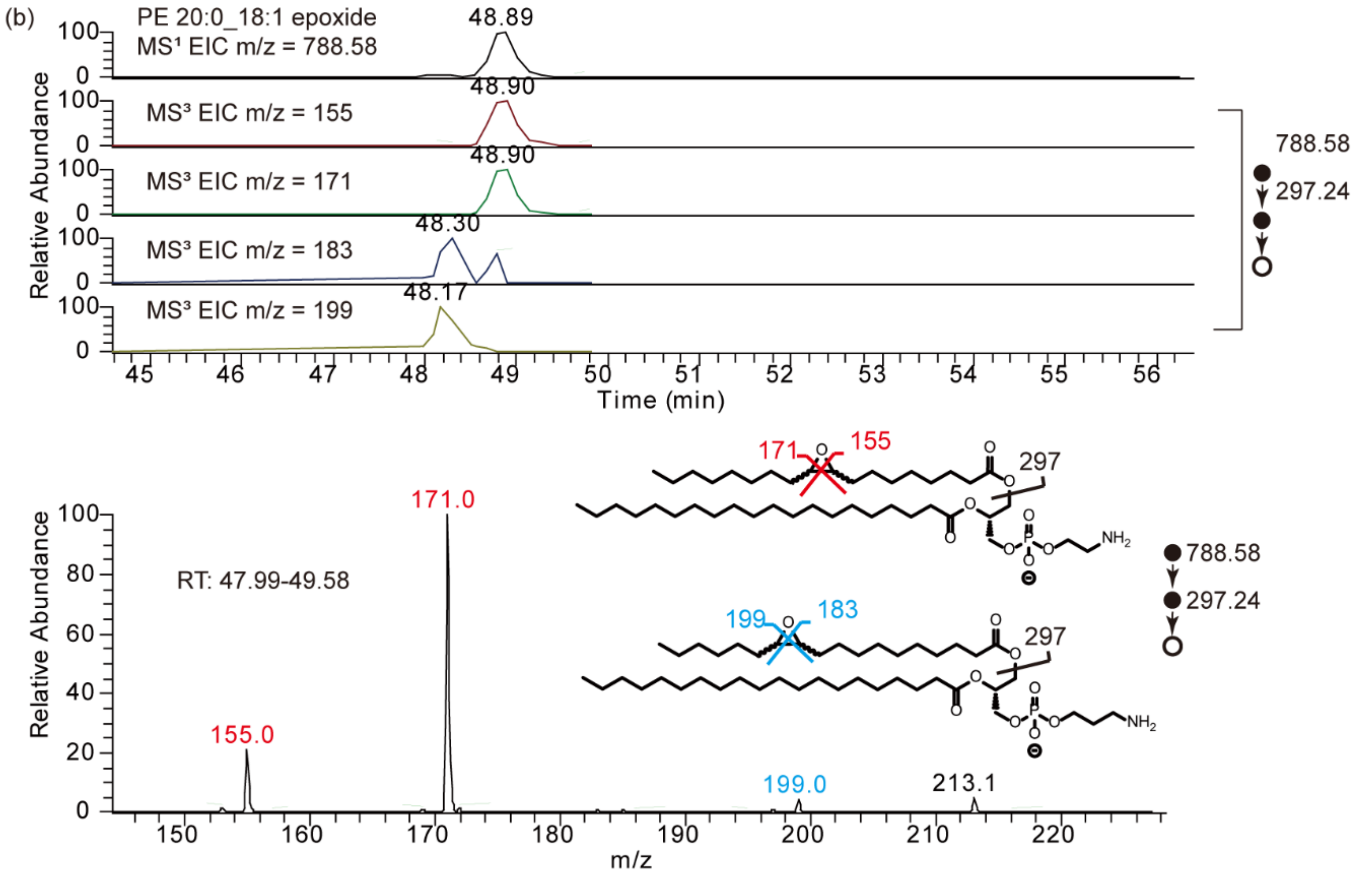
(b)
PE 20:0_18:1 epoxide
MS¹ EIC m/z = 788.58
48.89
MS³ EIC m/z = 155
48.90
MS³ EIC m/z = 171
48.90
MS³ EIC m/z = 183
48.30
MS³ EIC m/z = 199
48.17
788.58
297.24
Relative Abundance
Time (min)
RT: 47.99-49.58
171.0
155.0
199.0
213.1
171
155
297
199
183
m/z

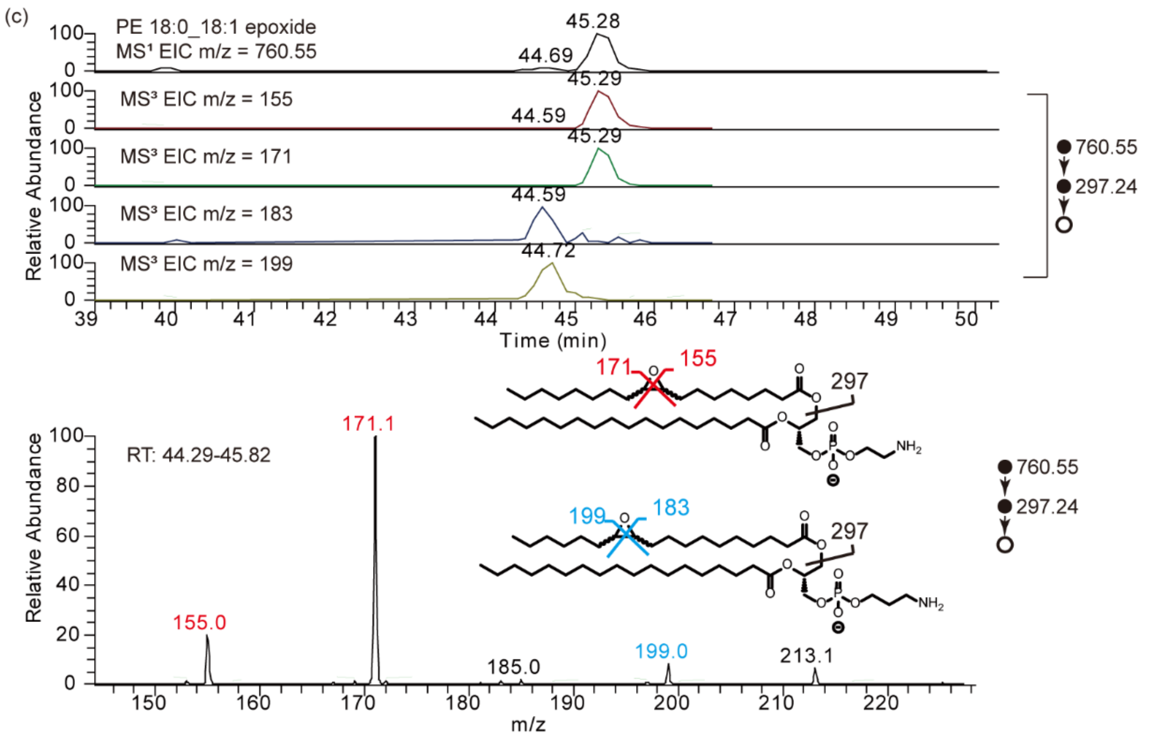

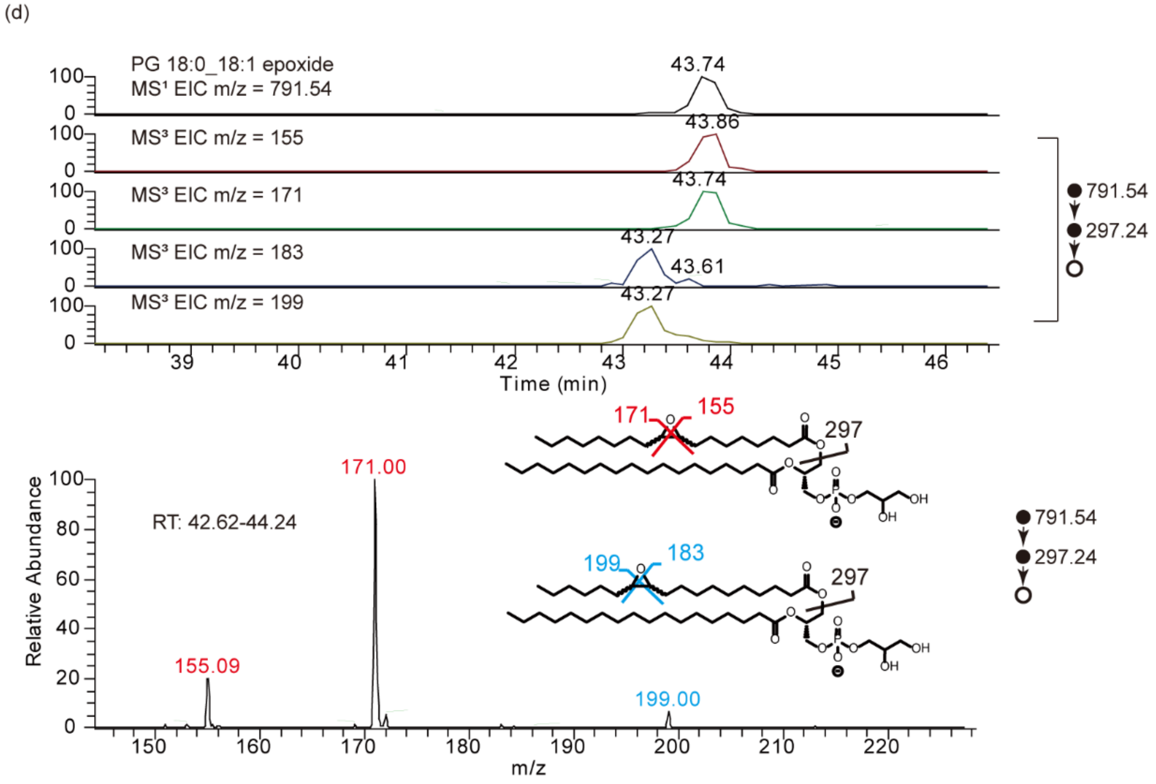

Figure. S7 The EIC and MS^3 spectra of the unsaturated phospholipid epoxide in the bacterial extracts from *B. longum* DSM20090 cultured in TSA. (a) PG 16:0_18:1 (b) PE 20:0_18:1 (c) PE 18:0_18:1 (d) PG 18:0_18:1

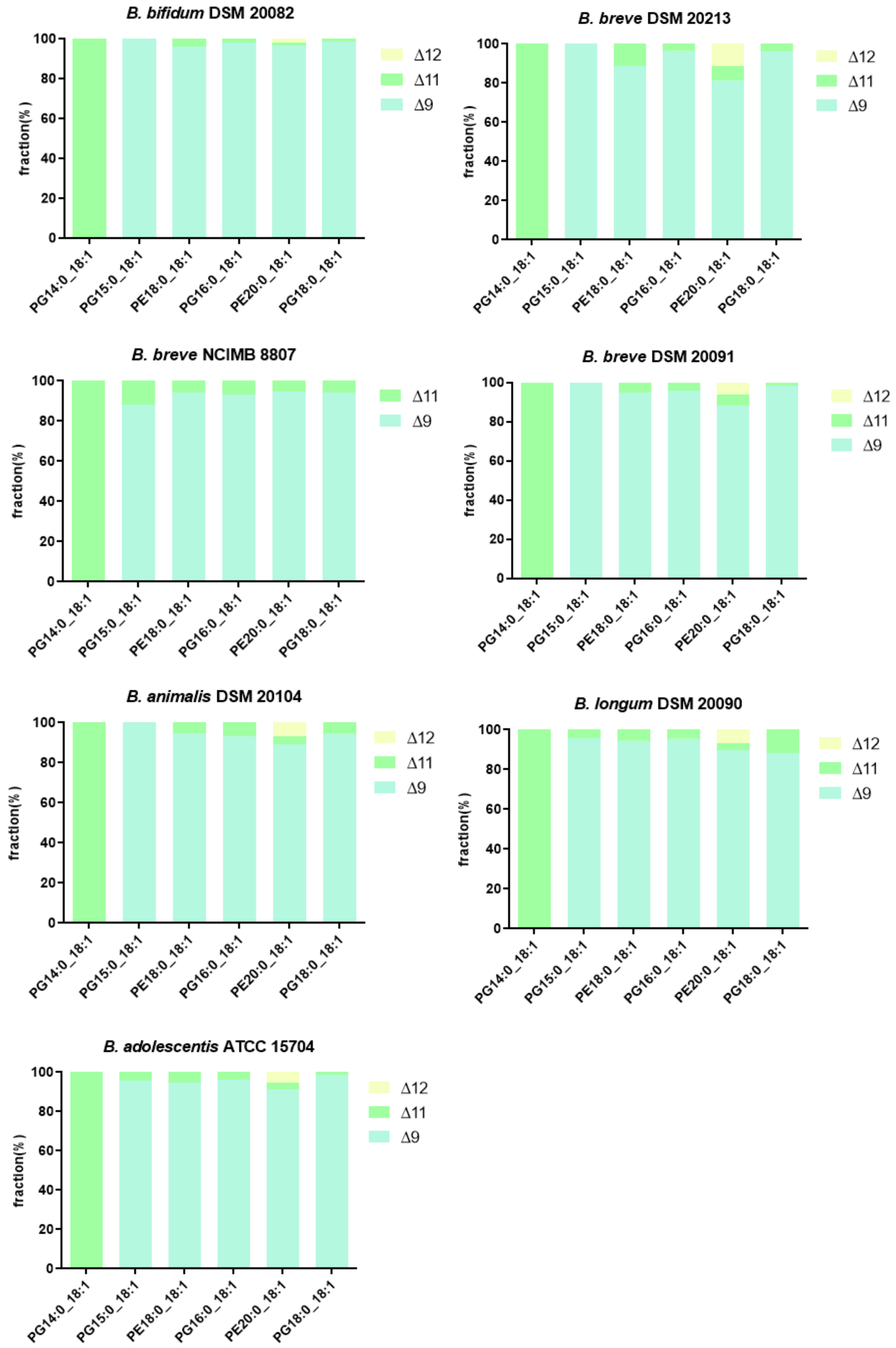

Figure. S8 Phospholipids C=C positional isomer profiling of the lipid extracts from the gut bacteria cultured in TSA agar

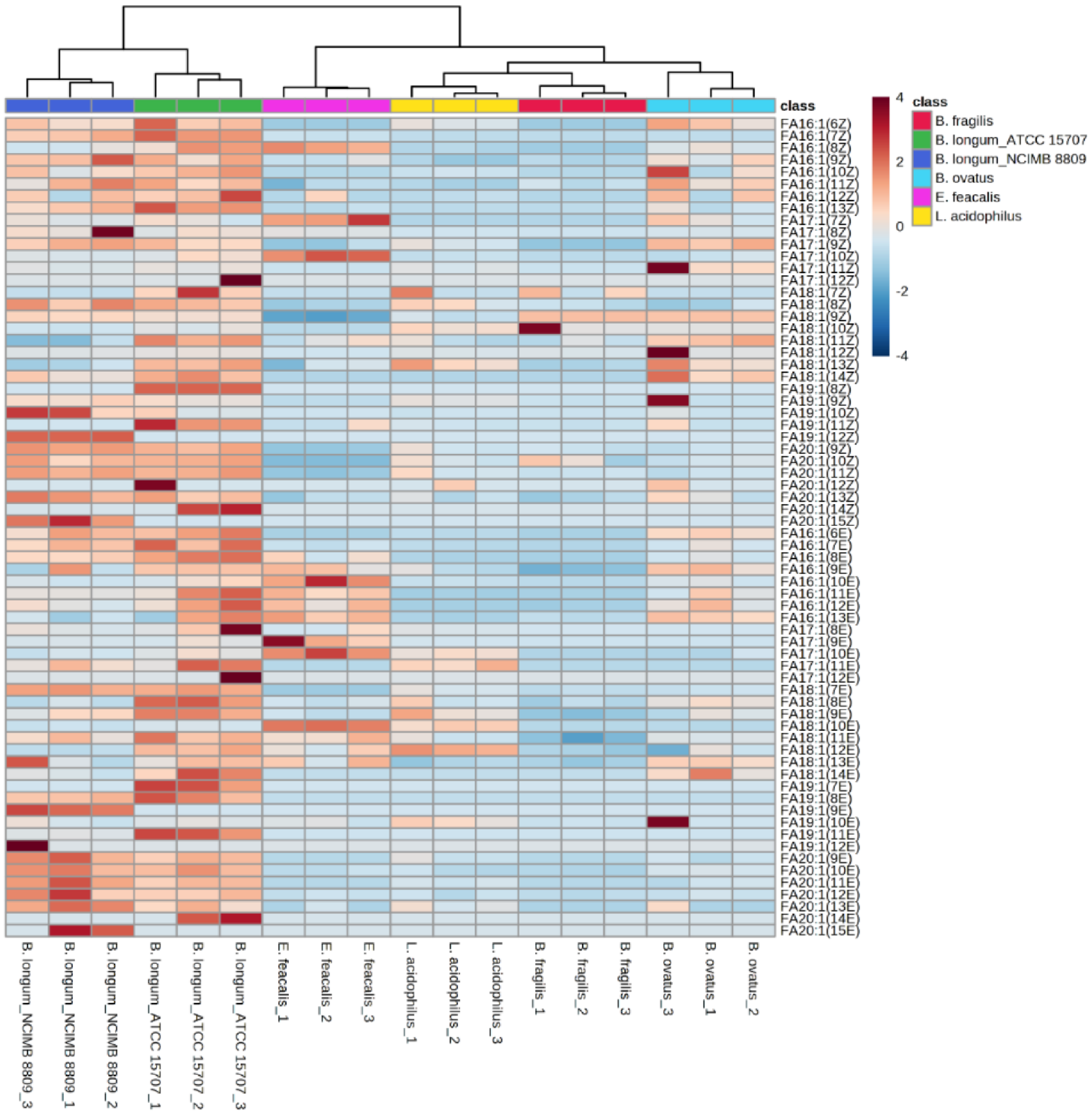

Figure. S9 Heatmap of the C=C isomers for 6 gut bacteria.

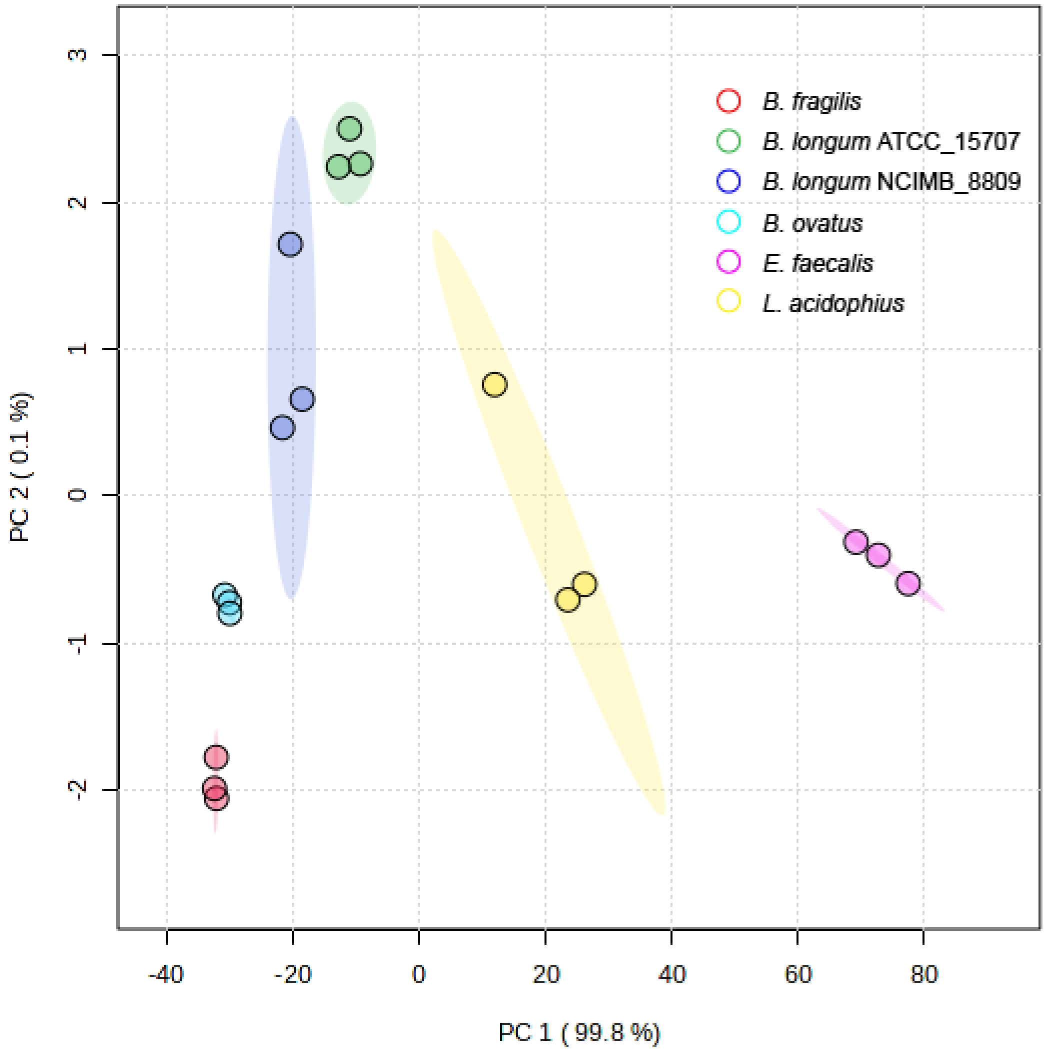

Figure. S10 PCA analysis of gut bacterial lipid extracts.

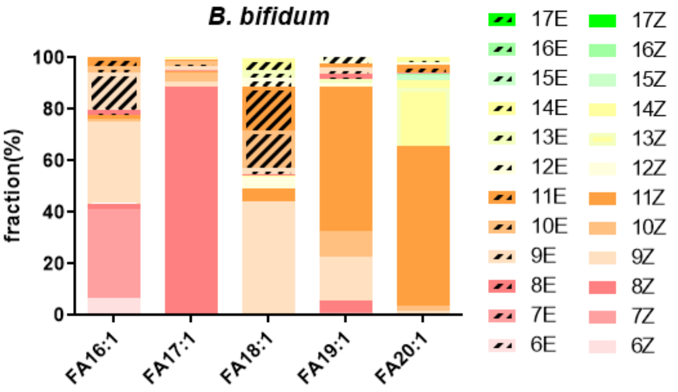
B. bifidum
fraction(%)
0
20
40
60
80
100
FA16:1
FA17:1
FA18:1
FA19:1
FA20:1
17E 17Z
16E 16Z
15E 15Z
14E 14Z
13E 13Z
12E 12Z
11E 11Z
10E 10Z
9E 9Z
8E 8Z
7E 7Z
6E 6Z

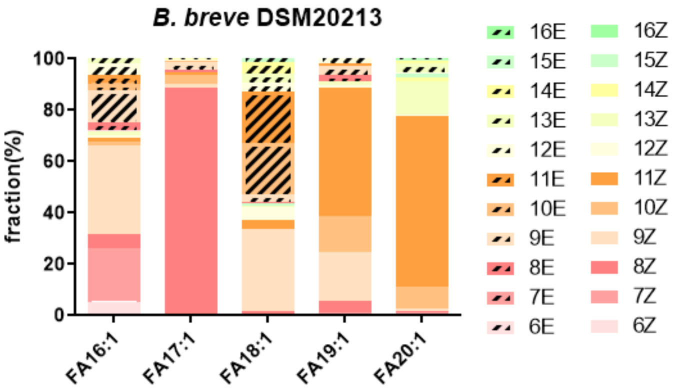
B. breve DSM20213
fraction(%)
0
20
40
60
80
100
FA16:1
FA17:1
FA18:1
FA19:1
FA20:1
16E 16Z
15E 15Z
14E 14Z
13E 13Z
12E 12Z
11E 11Z
10E 10Z
9E 9Z
8E 8Z
7E 7Z
6E 6Z

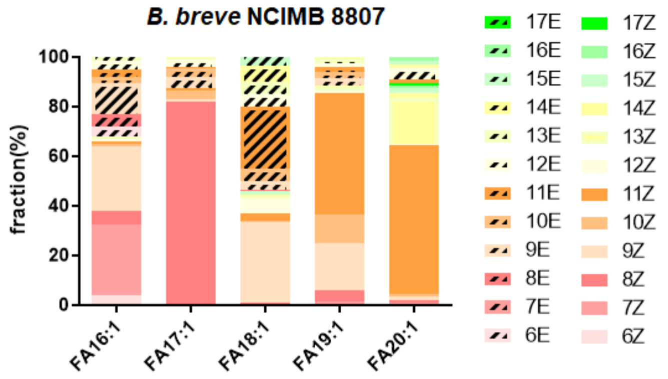
B. breve NCIMB 8807
fraction(%)
0
20
40
60
80
100
FA16:1
FA17:1
FA18:1
FA19:1
FA20:1
17E 17Z
16E 16Z
15E 15Z
14E 14Z
13E 13Z
12E 12Z
11E 11Z
10E 10Z
9E 9Z
8E 8Z
7E 7Z
6E 6Z

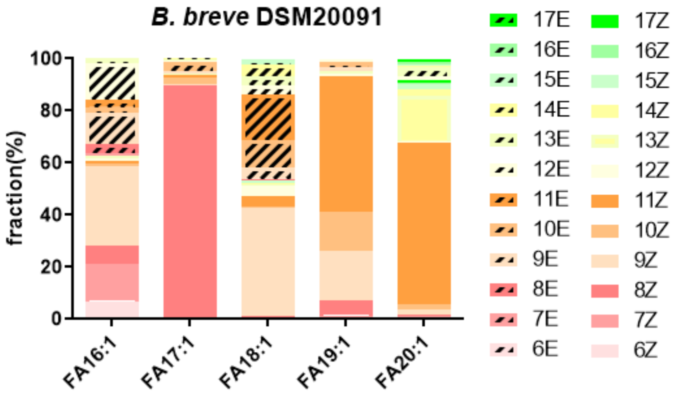
B. breve DSM20091
fraction(%)
0
20
40
60
80
100
FA16:1
FA17:1
FA18:1
FA19:1
FA20:1
17E 17Z
16E 16Z
15E 15Z
14E 14Z
13E 13Z
12E 12Z
11E 11Z
10E 10Z
9E 9Z
8E 8Z
7E 7Z
6E 6Z

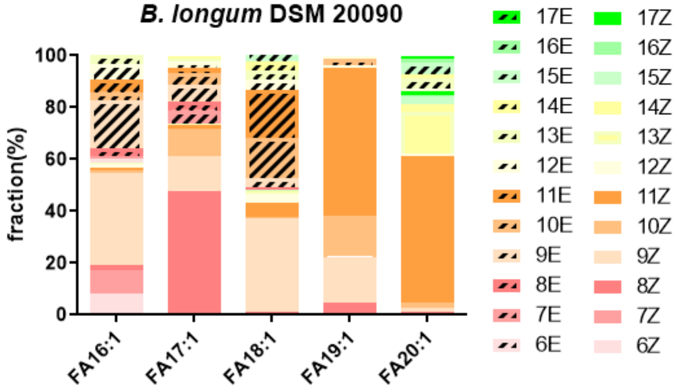
B. longum DSM 20090
fraction(%)
0
20
40
60
80
100
FA16:1
FA17:1
FA18:1
FA19:1
FA20:1
17E 17Z
16E 16Z
15E 15Z
14E 14Z
13E 13Z
12E 12Z
11E 11Z
10E 10Z
9E 9Z
8E 8Z
7E 7Z
6E 6Z

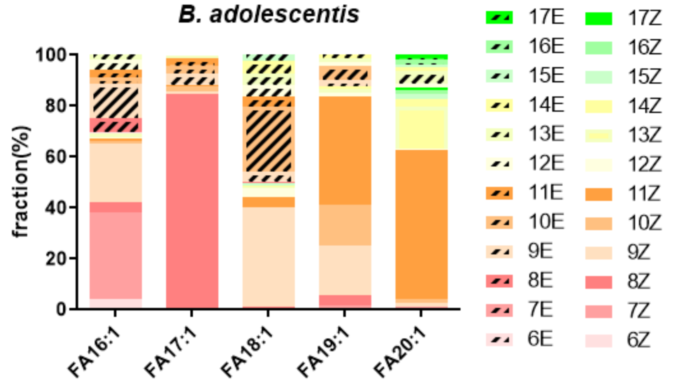
B. adolescentis
fraction(%)
0
20
40
60
80
100
FA16:1
FA17:1
FA18:1
FA19:1
FA20:1
17E 17Z
16E 16Z
15E 15Z
14E 14Z
13E 13Z
12E 12Z
11E 11Z
10E 10Z
9E 9Z
8E 8Z
7E 7Z
6E 6Z

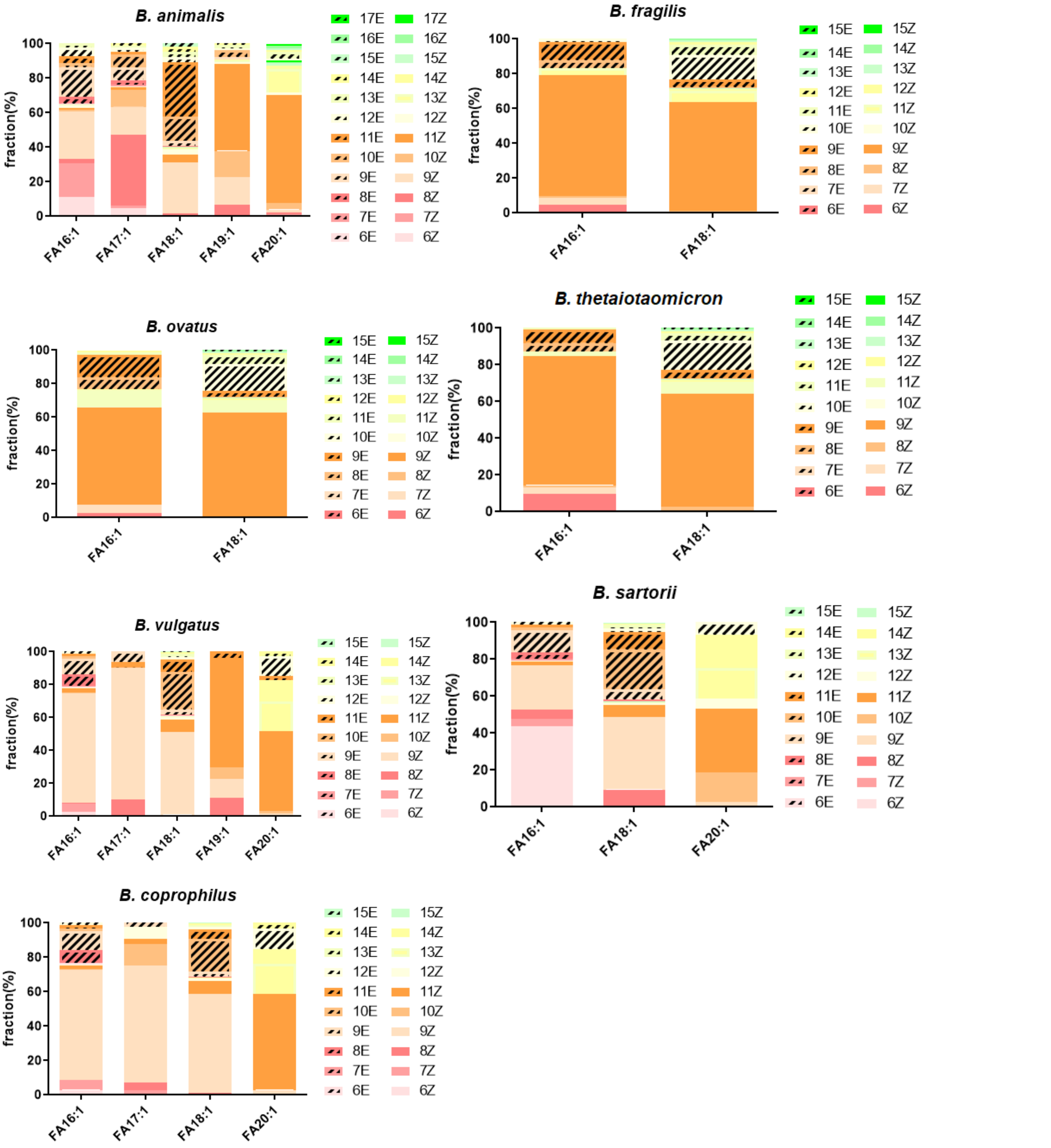

Figure. S11 FA 16:1-FA 20:1 C=C isomer profiling of the lipid extracts from the gut bacteria cultured in TSA agar.

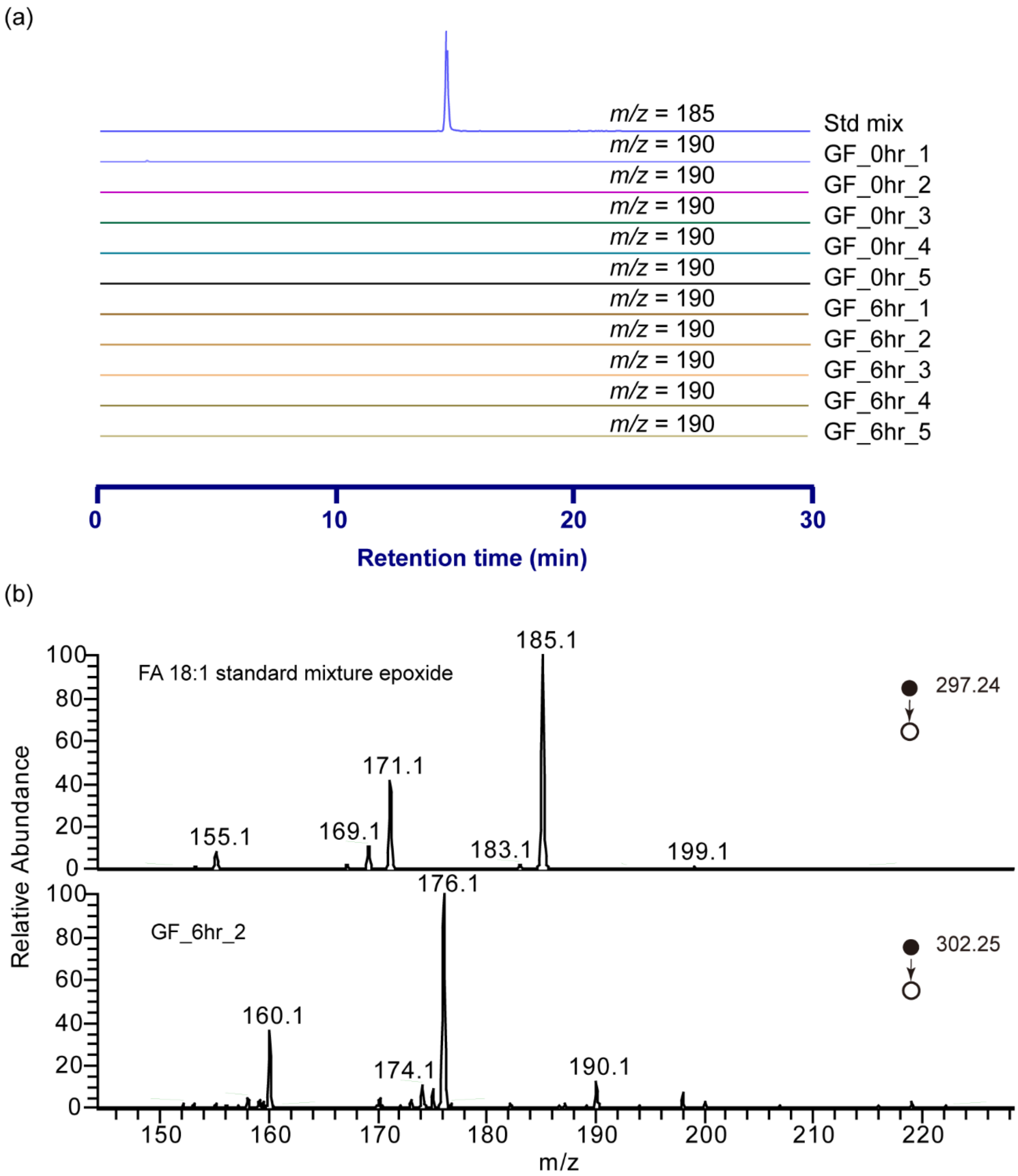

Figure. S12 *In vivo* isotope tracking with germ-free mice. (a) MS^2 EICs of the diagnostic ions for FA 18:1 10E. (b) MS^2 spectra of FA 18:1 epoxide.

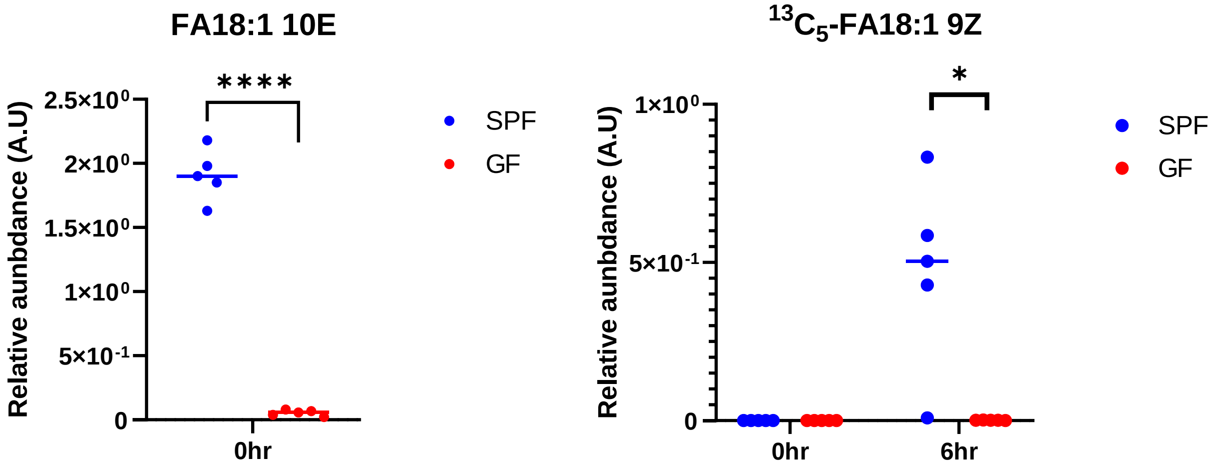

Figure. S13 Quantification of FA 18:1 10E & $^{13}C_5$-FA18:1 9Z in the SPF mice feces and GF mice feces.
* : p-value < 0.05
